# An immunoregulator nanomedicine approach for the treatment of tuberculosis

**DOI:** 10.3389/fbioe.2023.1095926

**Published:** 2023-05-25

**Authors:** Luona Yang, Lee Chaves, Hilliard L. Kutscher, Shanta Karki, Maria Tamblin, Patrick Kenney, Jessica L. Reynolds

**Affiliations:** ^1^ Department of Medicine, Jacobs School of Medicine and Biomedical Sciences, University at Buffalo, Buffalo, NY, United States; ^2^ Department of Neurosurgery, Jacobs School of Medicine and Biomedical Sciences, University at Buffalo, Buffalo, NY, United States; ^3^ Department of Pediatrics, Jacobs School of Medicine and Biomedical Sciences, University at Buffalo, Buffalo, NY, United States

**Keywords:** nanoparticles, immunostimulatory, tuberculosis, beta-glucan, macrophage.

## Abstract

**Introduction:** A nanoparticle composed of a poly (lactic-co-glycolic acid) (PLGA) core and a chitosan (CS) shell with surface-adsorbed 1,3 β-glucan (β-glucan) was synthesized. The exposure response of CS-PLGA nanoparticles (0.1 mg/mL) with surface-bound β-glucan at 0, 5, 10, 15, 20, or 25 ng or free β-glucan at 5, 10, 15, 20, or 25 ng/mL in macrophage *in vitro* and *in vivo* was investigated.

**Results:** *In vitro* studies demonstrate that gene expression for IL-1β, IL-6, and TNFα increased at 10 and 15 ng surface-bound β-glucan on CS-PLGA nanoparticles (0.1 mg/mL) and at 20 and 25 ng/mL of free β-glucan both at 24 h and 48 h. Secretion of TNFα protein and ROS production increased at 5, 10, 15, and 20 ng surface-bound β-glucan on CS-PLGA nanoparticles and at 20 and 25 ng/mL of free β-glucan at 24 h. Laminarin, a Dectin-1 antagonist, prevented the increase in cytokine gene expression induced by CS-PLGA nanoparticles with surface-bound β-glucan at 10 and 15 ng, indicating a Dectin-1 receptor mechanism. Efficacy studies showed a significant reduction in intracellular accumulation of *mycobacterium tuberculosis* (*Mtb*) in monocyte-derived macrophages (MDM) incubated with on CS-PLGA (0.1 mg/ml) nanoparticles with 5, 10, and 15 ng surface-bound β-glucan or with 10 and 15 ng/mL of free β-glucan. β-glucan-CS-PLGA nanoparticles inhibited intracellular *Mtb* growth more than free β-glucan alone supporting the role of β-glucan-CS-PLGA nanoparticles as stronger adjuvants than free β-glucan. *In vivo* studies demonstrate that oropharyngeal aspiration (OPA) of CS-PLGA nanoparticles with nanogram concentrations of surface-bound β-glucan or free β-glucan increased TNFα gene expression in alveolar macrophages and TNFα protein secretion in bronchoalveolar lavage supernatants.

**Discussion:** Data also demonstrate no damage to the alveolar epithelium or changes in the murine sepsis score following exposure to β-glucan-CS-PLGA nanoparticles only, indicating safety and feasibility of this nanoparticle adjuvant platform to mice by OPA.

## Introduction

Tuberculosis (TB) is a disease caused by *Mycobacterium tuberculosis* (*Mtb*), which primarily affects the lungs (pulmonary TB) and is hosted by macrophages, particularly alveolar macrophages (Weiss and Schaible, 2015; Upadhyay et al., 2018). According to the World Health Organization, TB remains a significant public health concern, with 1.5 million people dying from TB in 2020, making it the 13th leading cause of death globally (WHO, 2021a; WHO, 2021b). In 2020, an estimated 10 million people became ill with TB, with over 95% of cases and deaths occurring in developing countries. It is worth noting that TB is currently the second leading infectious cause of death worldwide, ranking just behind COVID-19. Prior to the COVID-19 pandemic, TB was the leading cause of death from a single infectious agent, surpassing HIV/AIDS (WHO, 2021a; WHO, 2021b). These statistics underscore the continued urgency of addressing the TB crisis.

Active TB disease is treated with a combination of antibacterial medications for a period of 6–12 months. Guidelines from the WHO recommend a 2-month regimen of isoniazid (INH), rifampicin (RIF), pyrazinamide (PZA), and ethambutol (EMB), followed by a 4-month regimen of isoniazid and RIF (Treatment) (WHO, 2021a; WHO, 2021b). Recently (Carr et al., 2022), findings from a randomized controlled trial indicate that a 4-month regimen containing rifapentine (RPT), moxifloxacin (MOX), INH, and PZA was as effective as the therapy described above. The CDC now recommends the 4-month regimen as a treatment option for U.S. patients aged ≥12 years with active TB (drug-susceptible) as of February 2022. The main issues with current TB treatment are the long duration and multiple medications, which often leads to pill fatigue and non-adherence to treatment as well as severe adverse effects and systemic toxicities associated with the treatment regimen. Overall, failure to respond to conventional treatment therapies for TB results in prolonged illness, increased direct and indirect health costs (necessary second-line treatment for patients), and increased mortality. Despite the availability of effective and low-cost TB pharmacotherapy for the past 50 years, the emergence of multi-drug resistant Mtb strains has highlighted the need for better TB regimens. Due to these concerns, inhaled adjuvant therapies are being explored as they increase immune activation at the site of infection (Nainwal et al., 2022)

TB’s primary target and the central host cell is macrophage (Weiss and Schaible, 2015). Mtb disrupts the macrophage’s intracellular killing and antigen presentation pathways, eventually leading to TB illness. Once engulfed by the macrophage, Mtb inhibits macrophage activation and becomes resistant to clearance (Upadhyay et al., 2018). Mtb has developed several pathways of immune evasion that allow it to survive within the macrophage. Mtb blocks the maturation of the phagolysosome by decreasing phagosomal acidification, the fusion of phagosomes with lysosomes, and phagosomal acquisition of lysosomal markers and characteristics (Armstrong and Hart, 1971; Frehel et al., 1986; Barker and Patrick, 1997; Ramachandra et al., 2005; Liu et al., 2017; Shi et al., 2019). Mtb also prevents macrophage activation, inhibiting the production of host-protective cytokines including TNFα, IL-1, IL-6, IL-8, and IL-12. IL-12 promotes IFN-γ production by T cells, which is essential for eradicating the Mtb bacterium through the maturation of the phagosome and induction of intracellular ROS/RNS. Intracellular ROS production is also reduced, and ROS/RNS are detoxified. Priming of other cells of the immune system, such as T cells, are reduced as cytokine production and antigen presentation are reduced (Armstrong and Hart, 1971; Frehel et al., 1986; Barker and Patrick, 1997; MacMicking et al., 2003; Ramachandra et al., 2005; Liu et al., 2017; Shi et al., 2019; Zhai et al., 2019). Overall, these mechanisms by Mtb enhance bacterial survival.

β-1,3-D glucans (β-glucan), homopolymers of glucose, in both soluble and particulate form have been used as an immunoadjuvant therapy for many years (Vetvicka et al., 2020). β-glucan is ligand for Dectin-1 receptors on macrophages, where its binding increases phagocytosis and stimulates the innate immune system, resulting in the production of intracellular ROS/RNS and pro-inflammatory cytokines (Pedro et al., 2021). β-glucan stimulates the innate immune system, activates complement, and has anti-infection and anti-tumor actions (Riggi and Di Luzio, 1961). β-glucan have been shown to protect against bacterial infection at microgram concentrations (Vetvicka et al., 2020). Previous studies have examined the effect of β-glucan on Mtb growth in macrophage, with varying results. β-glucan at microgram concentrations reduced the survival of Mtb H37Rv in peritoneal macrophages from BALB/c mice (Hetland and Sandven, 2002). Additionally, the number of Mtb colony-forming units (CFUs) was significantly decreased in β-glucan treated cells compared to control, demonstrating that β-glucan is anti-mycobacterial (Moorlag et al., 2020). In contrast, Betz et al., demonstrated that insoluble whole β-glucan and a soluble β-glucan extract did not inhibit intracellular Mtb in macrophage at microgram concentrations (Betz et al., 2011). Additional studies have shown that β-glucan can be formulated into microsphere Glucan particles (2–4 μm microspheres) and utilized as adjuvant in combination with vaccine delivery (Mirza et al., 2017).

Based on the interaction between the ligand β-glucan and the receptor Dectin-1 expressed on macrophages, as well as findings from previous studies (Hetland and Sandven, 2002; Betz et al., 2011; Dube et al., 2014; Kutscher et al., 2019) we developed a macrophage-targeted nanoparticle that utilizes β-glucan as a surface-targeting ligand. In a previous study, we demonstrated that CS-PLGA nanoparticles with 25 ng suface bound β-glucan significantly enhanced *in vitro* alveolar like macrophage secretion of cytokines and reactive oxygen species (ROS), providing proof of concept for this nanoparticle as an adjuvant (Dube et al., 2014). In further developing this platform the primary objective of the current study was to determine the exposure-response relationships between pro-inflammatory cytokines and ROS responses to the amount of β-glucan surface adsorbed to a biodegradable nanoparticle both *in vitro* and *in vivo*. Moreover, we sought to determine if using significantly lower amounts of β-glucan compared to previous studies by Hetland and Sandven (2002) and Betz et al. (2011) that utilized microgram concentrations of β-glucan, would also elicit biological mediators and reduce intracellular *Mtb* in macrophages. In addition, while Hetland and Sandven (2002) and Betz et al. (2011) studies utilized soluble or microparticulate forms of β-glucan, this study used β-glucan surface-adsorbed to a nanoparticle. The therapeutic potential of this surface-adsorbed β-glucan CS-PLGA adjuvant therapy has yet to be determined. The secondary objective of this study was to identify safe and effective exposures following the administration of our nanoparticulate in dosage form, both *in vitro* and *in vivo*. This is the first study to give Glu-CS-PLGA nanoparticle or free β-glucan by oropharyngeal aspiration (OPA) to mice and determine the direct effects on alveolar macrophage activation and potential adjuvant therapy that activates the innate immune response when given locally.

For the first time, we demonstrate that inhaled Glu-CS-PLGA nanoparticles stimulate the host-immune response while being safe (*in vivo*) and reduce *Mtb* in cultured macrophage (*in vitro*). This study is focused on developing an adjunct therapy that stimulates the host-immune response rather than focusing on drug delivery to avoid or limit drug-resistance. The combined findings from this study will inform future preclinical study designs for adjuvant and drug delivery studies using this nanoparticle system. Future studies will evaluate the *in vivo* drug delivery ability of this nanoparticles system based on optimized ligand results from this study.

## Materials and methods

### Glu-CS-PLGA nanoparticles

#### Materials

Poly (lactic-co-glycolic acid) (PLGA) (lactide:glycolide = 50:50, molecular weight (MW) = 38–54 kDa), poly (vinyl alcohol) (PVA) (Mowiol^®^4–88, MW = 31 kDa), chitosan oligosaccharide lactate (CS) (MW = 5 kDa), 1,3-β-glucan (β-glucan or Glu when bound to nanoparticle) and Cy5 were purchased from Millipore-Sigma Aldrich (St. Louis, MO). All reagents were used as received without further purification.

#### Synthesis

Nanoparticles were synthesized using nanoprecipitation with solvent evaporation. PLGA (20 mg) and Cy5 (18.5 μg) were dissolved in acetone (1 mL) and added dropwise to 10 mL of 0.5% PVA stirred at 700 RPM in a round bottom flask. The acetone phase was dispensed using a syringe pump at rate of 0.3 mL/min. The resulting nanoprecipitation was stirred 30 min under 4 L/min air to remove the acetone. An aliquot was removed to measure size and zeta potential. To the resulting nanosuspension, 500 μL of 3 mg/mL CS and 250 μL of 100 mM sodium acetate buffer pH 4.4 were added to form the shell and stirred for 5 min. An aliquot was removed to measure size and zeta potential. The nanoparticle suspension was washed once with water by centrifugation (16,100 x g, 20 min) and re-suspended in PBS. Surface bound β-glucan (5, 10, 15, 20, or 25 ng prepared as 5 μg/mL in 5 mM NaOH and PBS) was added to the 0.1 mg/mL CS-PLGA nanoparticle suspension and incubated for 5 min before centrifugation (16,100 x g, 10 min) and re-suspension in PBS at 2 mg CS-PLGA/ml (*in vitro*) or 20 mg/mL CS-PLGA (*in vivo*) (Kutscher et al., 2019). The content of surface β-glucan by electrostatic binding was determined using Glucatell kit (Associate of Cape Cod, East Falmouth, MA) (Neun et al., 2020). Glu-CS-PLGA nanoparticle size, polydispersity index, and zeta potential were determined by dynamic light scattering (DLS) (Horiba nanoPartica SZ-100 series) (Dube et al., 2014; Kutscher et al., 2019).

#### Electron microscopy

Aliquots of nanoparticles were taken during fabrication. Samples were identically prepared on formvar/carbon film 200 mesh copper grids. Grids were glow discharged for 30 s to improve their hydrophilicity prior to adding 5 μL of nanoparticles suspension. Using filter paper, the sample was drawn off and the grids were rinsed with 5 μL of distilled water. Following the water rinse, 5 μL of 2% aqueous uranyl acetate was added to the grid and drawn off with filter paper as in the previous steps. The grids were imaged at University at Buffalo’s Electron Microscopy Core Lab (Jacobs School of Medicine and Biomedical Sciences, Buffalo, NY) on a Hitachi HT7800 transmission electron microscope operating at 100 kV and captured by a Gatan Rio 16 CMOS camera.

### THP-1 macrophage

#### Nanoparticle exposure

THP-1 (ATCC- TIB-202) is a monocytic cell line from a human leukemia patient that has been used extensively to research monocyte/macrophage functions, processes, signaling pathways, and nutrition and drug transport. This cell line is a popular model for estimating monocyte and macrophage activity regulation (Essaji et al., 2013; Chanput et al., 2014; Kutscher et al., 2019). THP-1 are cultured in complete media consisting of RPMI 1640, 10% fetal bovine serum, 1% Penicillin-Streptomycin, 0.05 mM 2-mercaptoethanol (ThermoFisher Scientific, Grand Island, NY). THP-1 cells were seeded in 6 well plates at a concentration of 1 × 10^6^ cells/mL in the presence of 100 nM PMA overnight to differentiate THP-1 monocytes to THP-1 macrophages (Essaji et al., 2013; Kutscher et al., 2019). Culture media was then changed, and cells were incubated for 4 days to complete differentiation. The cells were subsequently incubated for 24 or 48 h with either CS-PLGA nanoparticles (0.1 mg/mL) with surface-bound β-glucan at 0, 5, 10, 15, 20, or 25 ng or free β-glucan at 5, 10, 15, 20, or 25 ng/mL. The cell supernatant was collected for ELISA, ROS, and transfer experiments (described below). The cell pellet was collected for gene expression studies. In a separate study THP-1 macrophages were incubated with 10 μg/mL laminarin (Dectin-1 antagonist; Millipore Sigma-Aldrich) (Smith et al., 2018) for 1 h prior to incubation with CS-PLGA nanoparticles (0.1 mg/mL) with either 10 or 15 ng surface bound β-glucan for 24 h. The cell pellets were collected for RNA isolation.

#### Generation of macrophage conditioned medium

Conditioned macrophage medium is defined as cell supernatant collected from THP-1 macrophage that were incubated for 24 h with CS-PLGA nanoparticles (0.1 mg/mL) with surface-bound β-glucan at 0, 5, 10, 15, 20, or 25 ng.

#### Jurkat T cells (CD4+)

Jurkat cells are human T CD4^+^ lymphocyte cells that are used to study T cell signaling. Jurkat T cells are cultured in complete media consisting of RPMI 1640, 10% fetal bovine serum, and 1% Penicillin-Streptomycin (ThermoFisher Scientific). Jurkat cells were seeded in 6 well plates at a concentration of 1 × 10^6^ cells/mL. Cells were then incubated with CS-PLGA nanoparticles (0.1 mg/mL) with surface-bound β-glucan at 0, 5, 10, 15, 20, or 25 ng for 24 h or 48 h. The cell pellets were collected for RNA isolation.

#### Transfer experiments

In other experiments Jurkat cells were incubated with 5% of macrophage conditioned medium for 24 h (described above). The cell pellets were collected for RNA isolation.

#### Intracellular uptake of nanoparticles

After 24 h of incubation with nanoparticles, cells were rinsed 3x with PBS and then fixed in 4% paraformaldehyde at room temperature for 30 min. Cells were subsequently counter-stained with the nuclear stain 4′,6-diamidino-2-phenylindole (DAPI) and photographed using the EVOS^®^ FL Cell Imaging System (Thermo Fisher Scientific, Carlsbad, CA) at ×20 magnification. The light cube used to visualize DAPI staining has an excitation of 357/44 nm and emission of 447/60 nm. The light cube use to visualize Cy5 has an excitation of 635/618 nm and emission of 692/640 nm. Image J software (NIH) was used to was used to quantify intracellular relative fluorescence density (RFU).

#### Cell viability assay

The CyQUANT™ MTT Cell Viability assay (Thermo-Fisher Scientific) was used to assess cytotoxicity to Glu-CS-PLGA nanoparticles. Cells were incubated with CS-PLGA nanoparticles (0.1 mg/mL) with surface-bound β-glucan at 0, 5, 10, 15, 20, or 25 ng or free β-glucan at 5, 10, 15, 20, or 25 ng/mL. THP-1 cells were next treated for 3 h with the [3-(4,5-Dimethylthiazol-2-yl]-2,5-diphenyltetrazolium bromide, (MTT) reagent (Sigma-Aldrich, St. Louis, MO), followed by the addition of sodium dodecyl sulfate (SDS) to lyse the cells and solubilize the colored crystals. Solubilized cell suspensions were quantified using an ELISA plate reader (BioTeK Synergy LX multimode reader, BioTek-Agilent, Santa Clara, CA) at 570 nm, and formazan was used as an indication of the number of viable cells.

#### RNA isolation

Macrophage or T cell pellets were lysed with Trizol reagent according to the established technique. Briefly, cell or tissue was lysed in 1 mL of TRIzol. 200 μL of chloroform was added followed by centrifugation. The aqueous layer was transferred into another 1.5 mL Eppendorf tube. 500 μL isopropanol was added to aqueous phase and mix gently followed by centrifugation. Pellet was washed in 1 mL of 75% ethanol. Then recentrifuged at max speed for 10 min (Chomczynski and Sacchi, 2006). The final RNA pellet was dried and resuspended in RNAse free water, and the concentration of RNA was determined using a NanoDrop ND-1000 Spectrophotometer (NanoDrop Technologies, Wilmington DE) at 260 nm. The isolated RNA was stored at −80°C until used.

#### Reverse transcription-cDNA synthesis

RNA was converted to complementary DNA (cDNA) using SuperScript IV reverse transcriptase (Thermo Fisher Scientific) according to the manufacturer’s instructions. Briefly, 5 μg of RNA was incubated with 50 μm anchor oligo d(T)20, and 10 mM dNTP for 65°C for 5 min (primer annealing). Then 5X SSIV buffer, 100 mM DTT, RNAse Inhibitor and SuperScript IV Reverse Transcriptase (200 U/µL) were incubated at 55°C for 10 min (RT reaction). The reaction was then inactivated by incubation at 80°C for 10 min cDNA was stored at −70°C until used.

#### Quantitative-PCR (Q-PCR)

Relative abundance of each mRNA species was assessed using the Luna^®^ Universal QPCR Master Mix Protocol (#M3003) (New England Biolabs Ipswich, MA) to perform Q-PCR. Briefly, forward and reverse primers for specific genes, at a final concentration of 0.25 μM, were incubated with 100 ng of template cDNA and 1X Luna Universal qPCR Master Mix for the following steps: 60 s, 95°C for 1 cycle (initial denaturation step), 15 s, 95°C (denaturation step), 30 s, 60°C for 45 cycles (extension step), and 60°C–95°C (melt curve) (New England Biolabs). Differences in threshold cycle number (CT) were used to quantify the relative amount of PCR target contained within each tube. Relative mRNA species expression was quantitated and expressed as transcript accumulation index (TAI = 2−(ΔΔCT)), calculated using the comparative CT method (Bustin, 2002; Radonic et al., 2004; Reynolds et al., 2012a; Reynolds et al., 2012b). All values were normalized to the constitutive expression of the housekeeping gene, TFRC (human) or PPIA (mouse). Primers sequences are shown in Table 1.

**TABLE 1:** QPCR primer sequences.

Primer name	Primer sequences	Gene ID	Use
Human genes			
**TRFC**	***Forward primer***	7037	housekeeping gene
	5′- TCT TTG GAC ATG CTC ATC TGG—3′		
	***Reverse primer***		
	5′-CCT TCC ATA TTC CCA AAC AGC—3′		
**IL-1β**	***Forward primer***	3553	Macrophage studies
	5′- GGC CCT AAA CAG ATG AAG TGC—3′		
	***Reverse primer***		
	5′- TGG TCG GAG ATT CGT AG—3′		
**IL-6**	***Forward primer***	3569	Macrophage studies
	5′- TGG ATT CAA GGA GAC TTG C—3′		
	***Reverse primer***		
	5′- TGC AGG AAC TGG ATC AGG AC—3′		
**TNFα**	***Forward primer***	7124	Macrophage studies
	5′- CCT CAG CCT CTT CTC CTT CC—3′		
	***Reverse primer***		
	5′- TGA GGG TTT GCT ACA TGG—3′		
**CXCL10**	***Forward primer***	3627	T cell studies
	5′- GTG GCA TTC AAG GAG TAC CTC TC—3′		
	***Reverse primer***		
	5′- GCT CCC CTC TGG TTT TAA GGA G—3′		
**IL-10**	***Forward primer***	3586	T cell studies
	5′- AAT GAA GGA TCA GCT GGA CAA C—3′		
	***Reverse primer***		
	5′-ATC GAT GAC AGC GCC GTA G—3′		
**IFNγ**	***Forward primer***	3458	T cell studies
	5′- GAA TTG GAA AGA GCA GAG TGA CAG—3′		
	***Reverse primer***		
	5′- GCT TTT CGA AGT CAT CTYC GTT TC—3′		
**Mouse Genes**			
**PPIA**	***Forward primer***	268373	housekeeping gene
	5′- CCG AAC CGG GRA TAA AGG AAG—3′		
	***Reverse primer***		
	5′- GCA AAC AGC TCG AAG GAG AC—3′		
**TNFα**	***Forward primer***	21926	Alveolar macrophage/Lung studies
	5′- GGT GCC TAT GTC TCA GCC TCT T—3′		
	***Reverse primer***		
	5′- GCC​ATA​GAA​CTG​ATG​AGA​GGG​AG—3′		

#### TNFα ELISA

Culture supernatants (CS-PLGA nanoparticles (0.1 mg/mL) with surface-bound β-glucan at 0, 5, 10, 15, 20, or 25 ng or free β-glucan at 5, 10, 15, 20, or 25 ng/mL) were analyzed for TNFα using an ELISA kit according to the manufacturer’s instructions (KHC3011, Thermo-Fisher Scientific) (TNFα Human ELISA Kit–Thermo-Fisher Scientific). Briefly, a standard curve in the range of 0 pg/mL to 1,000 pg/mL of recombinant human (hu) TNFα was generated. 100 μL of standards or unknowns were added to each well and incubated for 2 h at room temperature. The plates were washed and 100 μL of Hu TNFα Biotin Conjugate solution was added to each well and incubated for 1 h at room temperature. The plate was washed and the 100 μL 1X Streptavidin-HRP solution was added to each well and incubated for 30 min at room temperature. The plates were washed and 100 μL stabilized chromogen was added to each well and incubated for 30 min at room temperature in the dark. 100 μL of Stop Solution was added to each well. The plates were read at 450 nm using a BioTeK Synergy LX multimode reader (BioTek-Agilent). The concentrations of each sample were detected based on optical density and the concentration of the standard.

#### Estimation of cellular reactive oxygen species (ROS)

Culture supernatants (CS-PLGA nanoparticles (0.1 mg/mL) with surface-bound β-glucan at 0, 5, 10, 15, 20, or 25 ng or free β-glucan at 5, 10, 15, 20, or 25 ng/mL) were analyzed for ROS quantification by OxiSelect *In Vitro* ROS/RNS assay kit (green fluorescence, STA-347) (Cell Biolabs, San Diego, CA, United States) according to the manufacture’s protocol (OxiSelect™ *in vitro* Ros/RNS assay kit (green fluorescence)—cell Biolabs). A standard curve in the range of 0 nM–10,000 nM of DCF was generated. In a 96-well plate, 50 μL of unknown sample was incubated with 50 μL of catalyst for 5 min at room temperature the supernatant was mixed with 2′,7′-dichlorofluorescin (DCFH) solution for 30 min at room temperature. A fluorescent plate reader (Synergy LX multimode reader (BioTek-Agilent) was used to measure the fluorescence intensity of DCF at 480 nm excitation and 530 nm emission. The concentrations of each sample were detected based fluorescence intensity and the concentration of the standard.

#### Efficacy studies-TB infection in monocyte-derived macrophage (MDM)

Blood donors were recruited at University at Buffalo; consents were obtained consistent with the policies of University at Buffalo Health Sciences Institutional Review Board (HSIRB). Whole blood samples from HIV-1 negative individuals were drawn in BD Vacutainer CPT™ tubes (Cell Preparation Tube with Sodium Heparin, BD Biosciences, Franklin Lakes, NJ). Peripheral blood mononuclear cells (PBMC) were separated by centrifugation (18–25°C, 1,500 rcf, 15 min) in CPT tubes, followed by wash with phosphate buffered saline (PBS) without calcium or magnesium. CD14^+^ cells (monocytes) were selected by plastic adherence (Reynolds et al., 2012a; Reynolds et al., 2012b). CD14^+^ cells were cultured in complete medium [RPMI 1640, 10% fetal bovine serum, 1% Penicillin-Streptomycin (Thermo Fisher Scientific), 10 ng/mL macrophage colony-stimulating factor (Millipore, Billerica, MA)] for 7 days for differentiation into monocyte-derived macrophage (MDM). MDM were used for *in vitro Mtb* infection. The attenuated *Mtb* strain H37Ra strain (ATCC-25177) expressing RFP protein was cultured Middlebrook 7H9 Broth with ADC Enrichment (BD Biosciences) at 37°C. MDM were infected with RFP-expressing *Mtb* (strain H37Ra) at TCID_50_ (tissue culture infectious dose) for 12 h. Cells were washed and then treated with CS-PLGA nanoparticles (0.1 mg/mL) with surface-bound β-glucan at 0, 5, 10 and 15 ng/mL or free β-glucan at 5, 10, and 15 ng/mL for 5 days. Cells were then harvested by trypsinization (Trypsin-EDTA 0.25%, 15 min) and fixed with BD Cytofix/Cytoperm (BD Biosciences) for 15 min. Cells were washed with PBS then analyzed by flow cytometry for % positive of RFP cells (indicative of presence of intracellular TB) using a 561 nm laser on BD Fortessa flow cytometer (BD Biosciences). Normalization was done based on cell number recovered, controls were set to 100%.

### *In vivo* studies

CD1 male mice, 30–35 g (Charles Rivers Laboratories) were housed under a 12 h normal phase light-dark cycle. Drinking water and food were freely available. All procedures involving animals were reviewed and approved by the Institutional Animal Care and Use Committee of the University at Buffalo.

#### Delivery by oropharyngeal aspiration (OPA)

Mice were anesthetized with 5% isoflurane. While anesthetized, mice were hung from a loop by their incisors, the tongue was pulled out with forceps, and a 50 μL dose of 20 mg/mL PLGA was administered to the distal part of the oropharynx until inhaled (Van Hoecke et al., 2017). Note: 20 mg GLU-CS-PLGA/mL = 1 mg GLU-CS-PLGA/50 μL dose = 31.25 μg GLU-CS-PLGA/Gram per body weight (gBW). The β-glucan was added to the same concentration of CS-PLGA nanoparticles, therefore all animals received 31.25 μg/gBW nanoparticles with a corresponding amount of 1.71, 3.43, 5.14, or 6.85 ng β-glucan/gBW. Free β-glucan was given at 1.71, 3.43, 5.14, or 6.85 ng β-glucan/gBW.

#### Effect of surface bound β-glucan on behavioral modifications in mice

The Murine Sepsis Score (MSS) rates on spontaneous activity, response to touch and auditory stimuli, posture, respiration rate and quality (labored breathing or gasping), and appearance. This is a validated MSS with high specificity and sensitivity for predicting the onset of severe sepsis and death during the experimental timeline (Shrum et al., 2014). Greater than 21, indicates sepsis (Shrum et al., 2014). CD1 mice were dosed with nanoparticles with 1.71, 3.43, 5.14, or 6.85 ng β-glucan gBW. Free β-glucan was given at 1.71, 3.43, 5.14, or 6.85 ng β-glucan/gBW. Prior to dose and immediately before euthanasia, a blind observer rated animals using the MSS. On Day 0, all animals scored a zero on the MSS.

#### Recovery of alveolar macrophage by bronchoalveolar lavage (BAL)

Serum was collected, and mice were euthanized at 24 h after OPA by cervical dislocation under 5% isoflurane anesthesia. Mice were pinned to a surgical board and a loop was place around the incisors of the mouse and pinned to secure the head. The mouse was dissected to expose the thoracic cavity and neck. Blunt dissection exposed the trachea and forceps were used to thread a silk suture under the trachea. A small hole was made in the trachea using a 20 G needle. A 20 G catheter was then placed in the hole on the trachea and then the suture was tied around the trachea and catheter to secure the catheter. A 5 mL syringe containing saline (0.9%) with a 3-way stopcock on the end was place it in the end of the catheter. 1 mL of saline was slowly injected to inflate the lung and removed into the second syringe. This was repeated 4 times, with an approximate recovery of 5 mL of saline/lung. The lavage fluid was then weighed and recorded (Alluri et al., 2017). The lavage fluid was centrifuged for 10 min at 500 x g at 4°C. Supernatant was analyzed for TNFα protein secretion using a murine TNFα ELISA (Mouse TNF-alpha DuoSet ELISA Catalog #: DY410; R&D Systems) per manufactures instructions. The BAL cell pellet was resuspended in Trizol for RNA isolation followed by Q-PCR (described above). Albumin in the BAL supernatant was quantitated using an albumin ELISA (Mouse Albumin ELISA Kit (ab108791)) per manufactures instructions (data not shown). After BAL, mouse lungs were extracted and snap frozen in dry ice. Lung tissue using a tissue homogenizer was lysed with Trizol reagent according to the established technique (Rio et al., 2010) and analyzed for cytokine expression by Q-PCR (data not shown). Serum was analyzed for TNFα protein secretion using a murine TNFα ELISA (data not shown).

#### Statistical analysis

Analysis was performed using GraphPad Prism, version 9.3.1 (GraphPad Software, San Diego, CA). For comparisons of every mean to a control mean, statistical analysis was done using ordinary one-way ANOVA followed by Dunnett’s multiple comparisons test. For comparisons of every group mean with every other group mean, statistical analysis was done using ordinary one-way ANOVA followed by Tukey’s multiple comparisons test. Data shown represent mean ± SEM. *p≤ 0.05; **p≤ 0.01; ***p≤ 0.001; ****p≤ 0.0001.

## Results

### Nanoparticle characterization

**FIGURE 1:**
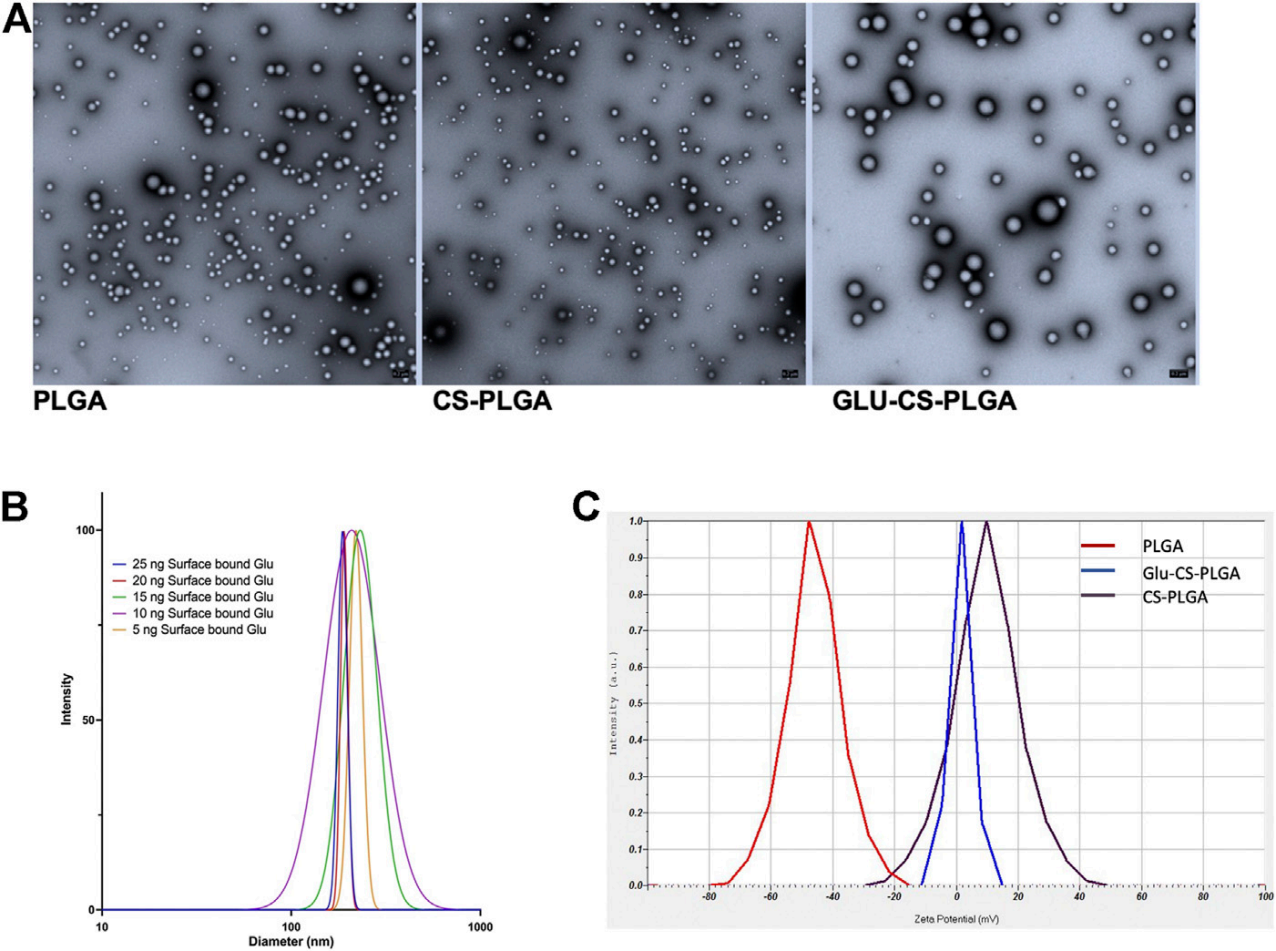
Characterization of Nanoparticles. **(A)** Representative TEM image of nanoparticles: PLGA-nanoparticles; CS-PLGA nanoparticles; Glu-CS-PLGA nanoparticles. **(B)** Size of CS-PLGA nanoparticles (0.1 mg/mL) with surface-bound β-glucan at 0, 5, 10, 15, 20, or 25 ng determined by DLS (*n* = 4). **(C)** Zeta potential comparison: red line: PLGA nanoparticles; brown line CS-PLGA nanoparticles; blue line: Glu-CS PLGA nanoparticles.

CS-PLGA nanoparticles (0.1 mg/mL) with surface-bound β-glucan at 0, 5, 10, 15, 20, or 25 ng were ∼217 nm in size determined by TEM and DLS (Figures 1A, B). The average zeta potential was +0.3 mV and was independent of β-glucan concentration. The uncoated PLGA nanoparticles containing Cy5 were ∼204 nm in diameter determined by DLS and had a zeta potential of −46 mV. After adding the CS shell, the diameter was ∼217 nm with a zeta potential of +12 mV due to the cationic nature of the CS shell. After adding β-glucan, the zeta potential was +0.3 mV (Figure 1C).

#### Intracellular uptake in macrophage

THP-1 macrophages were exposed to CS-PLGA nanoparticles (0.1 mg/mL) with surface-bound β-glucan at 0, 5, 10, 15, 20, or 25 ng to assess how modifying the concentration of the ligand (β-glucan) affected intracellular nanoparticle uptake. Data shown in Supplementary Figure S1A demonstrate representative images of THP-1 macrophage internalization of the nanoparticles following 24 h of exposure. Image analysis demonstrates that the concentration of the targeting ligand significantly modulated intracellular nanoparticle uptake (Supplementary Figure S1B). As surface bound β-glucan increases from 5 ng (11.79 ± 0.94 RFU; *p* = 0.0003), 10 ng (21.90 ± 0.82 RFU; *p* ≤ 0.0001), 15 ng (26.90 ± 2.30 RFU; *p* ≤ 0.0001) intracellular uptake is significant increased compared to control (1.52 ± 0.28 RFU); maximal uptake occurs at 15 ng. Intracellular uptake at 20 ng (21.31 ± 0.29 RFU; *p* ≤ 0.0001) and 25 ng (17.26 ± 0.65 RFU; *p* ≤ 0.0001) surface bound β-glucan is significantly increased compared to control (1.52 ± 0.28 RFU); yet significantly decreased compared to 15 ng (*p* = 0.036; *p* = 0.0006, respectively).

#### Cell viability assay for macrophage

To assess cellular viability, THP-1 macrophage were exposed for 24 or 48 h to either CS-PLGA nanoparticles (0.1 mg/mL) with surface-bound β-glucan at 0, 5, 10, 15, 20, or 25 ng or free β-glucan at 5, 10, 15, 20, or 25 ng/mL followed by a CyQUANT™ MTT Cell Viability Assay (Supplementary Figure S2). At 24 h, there was no significant change in macrophage viability observed with surface bound β-glucan at 5, 10, or 15 ng or free β-glucan at 5, 10, or 15 ng/mL compared to control. However, a significant decrease in cell viability occurred at nanoparticles with 20 ng (*p* ≤ 0.01) and 25 ng (*p* ≤ 0.01) of surface bound β-glucan and free β-glucan at 20 ng/mL (*p* ≤ 0.01) or 25 ng/mL (*p* ≤ 0.001) (Supplementary Figure S2A). Cellular viability after 48 h exposure to CS-PLGA nanoparticles (0.1 mg/mL) with surface-bound β-glucan at 0, 5, 10, 15, 20, or 25 ng or free β-glucan at 5, 10, 15, 20, or 25 ng/mL is shown in Supplementary Figure S2B, and results are similar to 24 h exposure. There was no significant change in macrophage viability at 5, 10, or 15 ng of surface bound β-glucan or 15 ng or free β-glucan at 5, 10, or 15 ng/mL compared to control. However, a significant decrease in cell viability occurred at nanoparticles with 20 ng (*p* ≤ 0.05) and 25 ng (*p* ≤ 0.01) of surface bound β-glucan and free β-glucan at 20 ng/mL (*p* ≤ 0.01) or 25 ng/mL (*p* ≤ 0.001).

#### Modulation of cytokine gene expression

**FIGURE 2:**
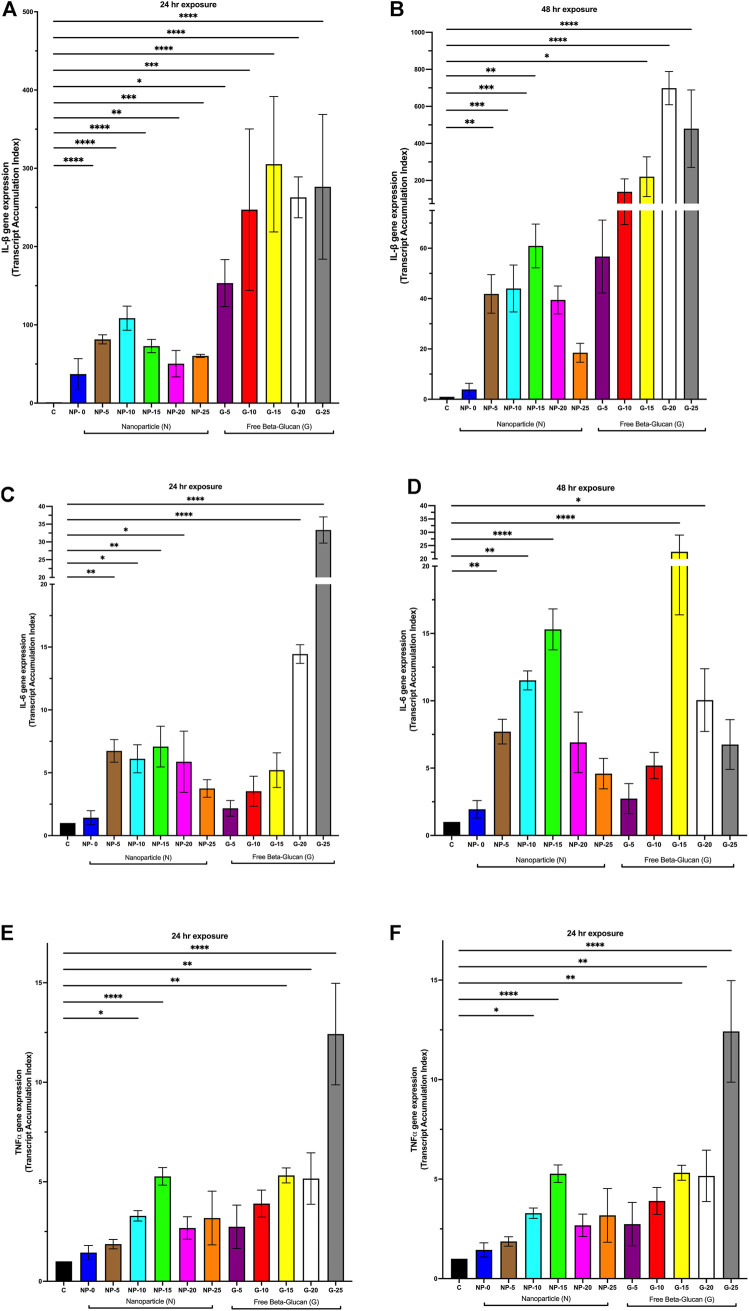
Effect of Increasing Amounts of Surface Bound β-glucan on Cytokine Gene Expression from Macrophage. CS-PLGA nanoparticles (0.1 mg/mL) with surface-bound β-glucan at 0, 5, 10, 15, 20, or 25 ng or free β-glucan at 5, 10, 15, 20, or 25 ng/mL were incubated with THP-1 macrophage for 24 h and 48 h; RNA was isolated and changes in gene expression were analyzed using Q-PCR. **(A)** IL-1β gene expression following 24 h exposure nanoparticles or free β-glucan (*n* = 3–8). **(B)** IL-1β gene expression following 48 h incubation with nanoparticles or free β-glucan (*n* = 3–7). **(C)** IL-6 gene expression following 24 h exposure to nanoparticles or free β-glucan (*n* = 3–11). **(D)** IL-6 gene expression following 48 h incubation with nanoparticles or free β-glucan (*n* = 3–7). **(E)** TNFα gene expression following 24 h exposure to nanoparticles or free β-glucan (*n* = 4–10). **(F)** TNFα gene expression following 48 h incubation with nanoparticles or free β-glucan (*n* = 4–6). Data shown represent the mean ± SEM. *p ≤ 0.05; **p ≤ 0.01; ***p≤ 0.001; ****p ≤ 0.0001; comparison to control. C = control; N = nanoparticle; G = β-glucan.

To further understand the role of β-glucan on the immunostimulatory properties of our nanoparticle, THP-1 macrophages were exposed for 24 or 48 h to either CS-PLGA nanoparticles (0.1 mg/mL) with surface-bound β-glucan at 0, 5, 10, 15, 20, or 25 ng or free β-glucan at 5, 10, 15, 20, or 25 ng/mL. Cytokine gene expression (IL-1β, IL-6, and TNFα) was next assessed using Q-PCR (Figure 2). At 24 h (Figure 2A), IL-1β gene expression was significantly increased following exposure to CS-PLGA nanoparticles (0.1 mg/mL) with 5 ng (81.39 ± 5.8 TAI; *p* ≤ 0.0001), 10 ng (108.50 ± 15.42 TAI; *p* ≤ 0.0001), 15 ng (72.88 ± 8.42 TAI; *p* ≤ 0.0001), 20 ng (50.43 ± 16.90 TAI; *p* ≤ 0.01), and 25 ng (60.29 ± 1.98 TAI; *p* ≤ 0.001) surface bound β-glucan compared to control (1.00 TAI). Free β-glucan significantly increased IL-1β gene expression at 5 ng (153.30 ± 30.05; *p* ≤ 0.05), 10 ng (247.10 ± 103.20 TAI, *p* ≤ 0.001), 15 ng (305.20 ± 86.55 TAI; *p* ≤ 0.0001), 20 ng (262.90 ± 26.09 TAI; *p* ≤ 0.0001), and 25 ng (276.30 ± 92.47; *p* ≤ 0.0001) surface bound β-glucan compared to control (1.00 TAI). (Figure 2A). At 48 h (Figure 2B), IL-1β gene expression was significantly increased following exposure to CS-PLGA nanoparticles (0.1 mg/mL) with 5 ng (41.85 ± 7.66 TAI; *p* ≤ 0.01), 10 ng (44.01 ± 9.32 TAI; *p* ≤ 0.001), 15 ng (60.91 ± 8.64 TAI; *p* ≤ 0.001), and 20 ng (39.46 ± 5.54 TAI; *p* ≤ 0.01) surface bound β-glucan compared to control (1.00 TAI). Free β-glucan significantly increased IL-1β gene expression 15 ng (220.40 ± 107.10 TAI; *p* ≤ 0.05), 20 ng (698.40 ± 89.62 TAI; *p* ≤ 0.0001), and 25 ng (479.70 ± 209.0; *p* ≤ 0.0001) surface bound β-glucan compared to control (1.00 TAI). (Figure 2B).

At 24 h (Figure 2C), IL-6 gene expression was significantly increased following exposure to CS-PLGA nanoparticles (0.1 mg/mL) with 5 ng (6.74 ± 0.95 TAI; *p* ≤ 0.01), 10 ng (6.11 ± 1.12 TAI; *p* ≤ 0.05), 15 ng (7.08 ± 1.60 TAI; *p* ≤ 0.01), and 20 ng (5.87 ± 2.44 TAI; *p* ≤ 0.05) of surface bound β-glucan compared to control (1.00 TAI). Free β-glucan significantly increased IL-6 gene expression at 20 ng (14.44 ± 0.79 TAI; *p* ≤ 0.0001) and 25 ng (33.35 ± 3.68 TAI; *p* ≤ 0.0001). At 48 h (Figure 2D), IL-6 gene expression was significantly increased following exposure to CS-PLGA nanoparticles (0.1 mg/mL) with 5 ng (7.70 ± 0.92 TAI; *p* ≤ 0.01), 10 ng (11.52 ± 0.71 TAI; *p* ≤ 0.01) and 15 ng (15.30 ± 1.52 TAI; *p* ≤ 0.0001) of surface bound β-glucan compared to control (1.00 TAI). Free β-glucan significantly increased IL-6 gene expression at 15 ng (22.66 ± 6.27 TAI; *p* ≤ 0.0001) and 20 ng (10.05 ± 2.37 TAI; *p* ≤ 0.05).

At 24 h (Figure 2E), TNFα gene expression was significantly increased following exposure to CS-PLGA nanoparticles (0.1 mg/mL) with 10 ng (3.28 ± 0.26 TAI; *p* ≤ 0.05) and 15 ng (5.27 ± 0.44 TAI; *p* ≤ 0.0001) with surface bound β-glucan compared to control (1.00 TAI). Free β-glucan significantly increased TNFα gene expression at 15 ng (5.32 ± 0.38 TAI; *p* ≤ 0.01), 20 ng (5.32 ± 1.29 TAI; *p* ≤ 0.01) and 25 ng (12.42 ± 2.55 TAI; *p* ≤ 0.0001). At 48 h (Figure 2F), TNFα gene expression was significantly increased following exposure to CS-PLGA nanoparticles (0.1 mg/mL) with 10 ng (4.92 ± 0.62 TAI; *p* ≤ 0.0001) and 15 ng (5.02 ± 0.36 TAI; *p* ≤ 0.0001), 20 ng (5.86 ± 0.75 TAI; *p* ≤ 0.0001), 25 ng (5.35 ± 0.69 TAI; *p* ≤ 0.0001) surface bound β-glucan compared to control (1.00 TAI). Free β-glucan significantly increased TNFα gene expression at 10 ng (3.46 ± 0.44 TAI; *p* ≤ 0.051), 15 ng (4.49 ± 0.23 TAI; *p* ≤ 0.001) and 20 ng (5.74 ± 0.19 TAI; *p* ≤ 0.0001).

#### Modulation of biological mediators

**FIGURE 3:**
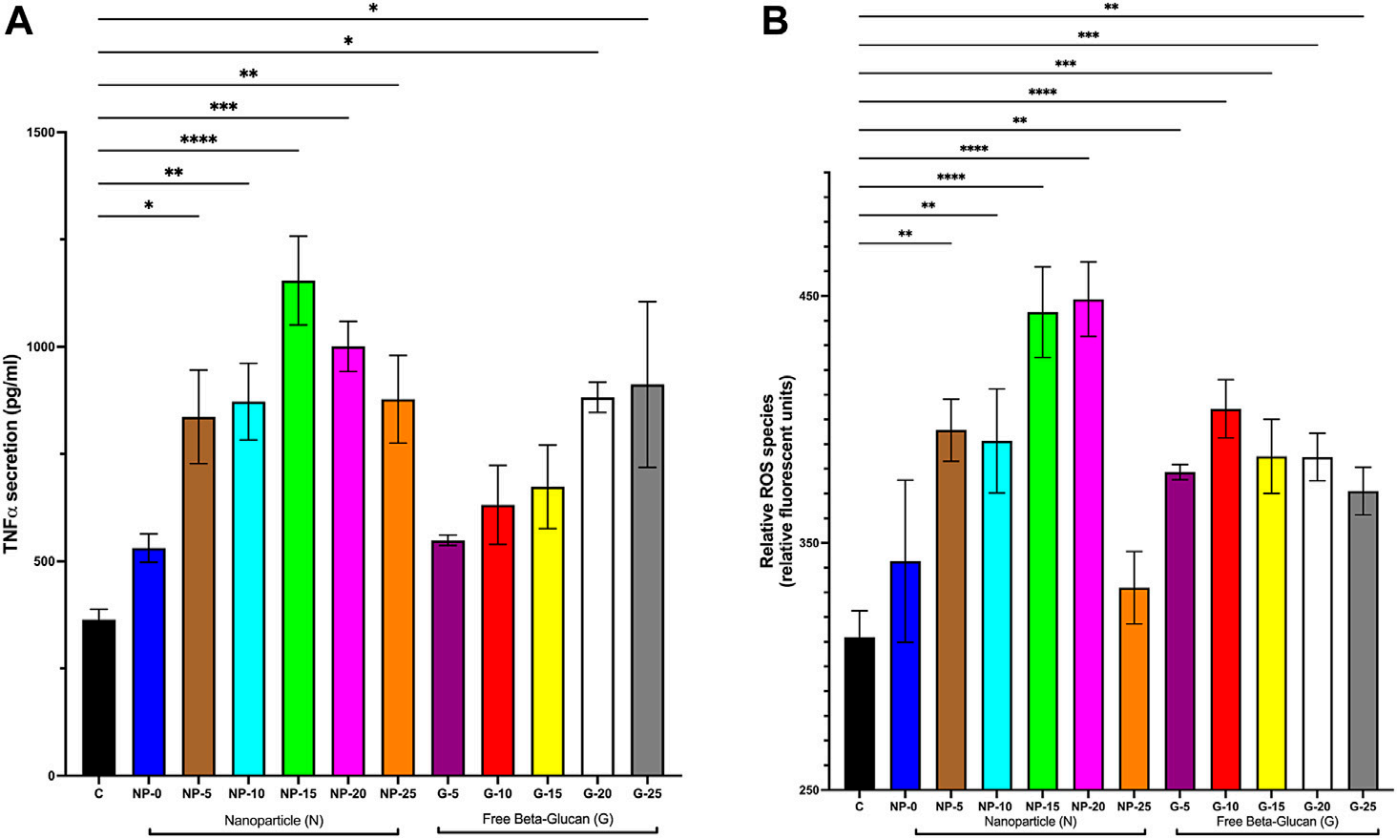
Effect of Increasing Amounts of Surface Bound β-glucan on biological mediators. CS-PLGA nanoparticles (0.1 mg/mL) with surface-bound β-glucan at 0, 5, 10, 15, 20, or 25 ng or free β-glucan at 5, 10, 15, 20, or 25 ng/mL were incubated with THP-1 macrophage for 24 h; cell supernatant was harvested and analyzed. **(A)** TNFα secretion was analyzed by ELISA (*n* = 4–11). **(B)** ROS production was analyzed by an assay specific to ROS (*n* = 4–9). Data shown represent the mean ± SEM. *p≤ 0.05; **p≤ 0.01; ***p≤ 0.001; ****p≤ 0.0001; comparison to control. C = control; N = nanoparticle; G = β-glucan.

To further investigate the immunostimulatory properties our nanoparticle, THP-1 macrophages were incubated for 24 h with either CS-PLGA nanoparticles (0.1 mg/mL) with surface-bound β-glucan at 0, 5, 10, 15, 20, or 25 ng or free β-glucan at 5, 10, 15, 20, or 25 ng/mL. Cell supernatant was extracted and evaluated for soluble TNFα by ELISA and ROS species production (Figure 3). TNFα secretion was significantly increased following exposure to CS-PLGA nanoparticles (0.1 mg/mL) with 5 ng (836.60 ± 109.10 pg/mL; *p* ≤ 0.05), 10 ng (878.8 ± 89.32 pg/mL; *p* ≤ 0.01), 15 ng (1,154 ± 103.50 pg/mL; *p* ≤ 0.0001), 20 ng (1,001 ± 58.34 pg/mL; *p* ≤ 0.001), and 25 ng (877.60 ± 102.20 pg/mL; *p* ≤ 0.01) surface bound β-glucan compared to control (363.70 ± 24.48 pg/mL) (Figure 3A). Free β-glucan significantly TNFα secretion increased at 20 ng (882.10 ± 35.20 pg/mL; *p* ≤ 0.05) and 25 ng (911 ± 193.1 pg/mL; *p* ≤ 0.05). ROS production was significantly increased following exposure to CS-PLGA nanoparticles (0.1 mg/mL) with 5 ng (395.70 ± 12.57 RFU; *p* ≤ 0.01), 10 ng (391.30 ± 21.05 RFU; *p* ≤ 0.01), 15 ng (443.50 ± 18.36 RFU; *p* ≤ 0.0001), and 20 ng (448.70 ± 15.05 RFU; *p* ≤ 0.0001) surface bound β-glucan compared to control (311.90 ± 10.66 RFU) (Figure 3B). CS-PLGA nanoparticles (0.1 mg/mL) with 0 ng of surface bound β-glucan had no effect on ROS production. Free β-glucan significantly increased ROS production at 5 ng (378.70 ± 3.07 RFU; *p* ≤ 0.01), 10 ng (404.30 ± 11.81 RFU; *p* ≤ 0.0001), 15 ng (385.10 ± 14.97 RFU; *p* ≤ 0.001), 20 ng (384.80 ng/mL ± 9.63; *p* ≤ 0.001) and 25 ng (371.00 ng/mL ± 9.66; *p* ≤ 0.01).

#### Receptor mediated effect

**FIGURE 4:**
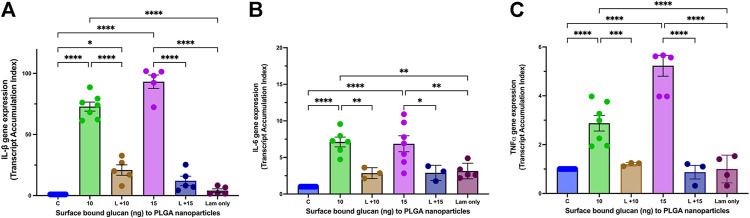
Effect of Laminarin on Glu-CS-PLGA Stimulated Cytokine Production in Macrophage. THP-1 macrophages were incubated with 10 μg/mL laminarin (Lam; Dectin-1 antagonist) for 1 h prior to incubation with CS-PLGA nanoparticles with either 10 ng or 15 ng surface bound β-glucan. RNA was isolated from macrophage 24 h later and changes in gene expression were analyzed using Q-PCR. **(A)** IL-1β gene expression (*n* = 6). **(B)** IL-6 gene expression (*n* = 6). **(C)** TNFα gene expression (*n* = 6). Data shown represent the mean ± SEM. *p≤ 0.05; **p≤ 0.01; ***p≤ 0.001; ****p≤ 0.0001, comparisons to control or comparisons to the mean of each column with the mean of every other column.

As β-glucan is a PAMP (pathogen associated molecular pattern) and binds to the PRR (pattern recognition receptor) Dectin-1, we next wanted to demonstrate that immunostimulatory effects of surface bound β-glucan on the nanoparticle were mediated by the Dectin-1 receptor on macrophage (Pedro et al., 2021). Based on results from our intracellular uptake and cellular viability studies, we tested only the 10 ng and 15 ng surface bound Glu-CS-PLGA nanoparticles in the presence of laminarin (Dectin-1 antagonist). Laminarin prevented the increase in IL-1β gene expression (Figure 4A) at 10 ng (20.71 ± 5.14 TAI; *p*≤ 0.0001 compared to 10 ng of β-glucan alone), 15 ng (12.11 ± 3.81 TAI; *p* ≤ 0.0001 compared to 15 ng of β-glucan alone) of surface bound β-glucan. Laminarin alone (4.09 ± 1.43 TAI; *p* = ns) did not increase in IL-1β gene expression. Regarding IL-6 gene expression (Figure 4B), laminarin also prevented the increase at 10 ng (2.86 ± 0.43 TAI; *p* = 0.009 compared to 10 ng), 15 ng (2.89 ± 0.60 TAI; *p* = 0.05 compared to 15 ng) of surface bound β-glucan. Laminarin alone (3.14 ± 0.48 TAI; *p* = ns) did not increase in IL-6 gene expression. Laminarin also prevented the increase in TNFα gene expression at 10 ng (1.20 ± 0.43 TAI; *p* ≤ 0.0001 compared to 10 ng) and 15 ng (0.89 ± 0.28 TAI; *p* ≤ 0.0001 compared to 15 ng) of surface bound β-glucan (Figure 4C). Laminarin alone (1.001 ± 0.28 TAI; *p* = ns) did not increase in TNFα gene expression.

#### Modulation of cytokine gene expression in Jurkat T cells

Since T cells are important cells in generating a complete immune response to TB, we explored the immunostimulatory properties of our nanoparticle in T cells. Jurkat were exposed to CS-PLGA nanoparticles (0.1 mg/mL) with surface-bound β-glucan at 0, 5, 10, 15, 20, or 25 ng for 24 or 48 h. Gene expression (CXCL10, IL-10, and IFNγ) was next assessed using Q-PCR (Supplementary Figure S3). No significant changes occurred in gene expression for CXCL10, IL-10, and IFNγ gene expression at 24 and 48 h (Supplementary Figures S3A–F), respectively. Since gene expression in T cells was not directly modulated by the β-glucan on the surface of the nanoparticle, we next studied the effects of conditioned macrophage medium on T cell gene expression. Conditioned macrophage medium is defined as cell supernatant collected from THP-1 macrophage that were incubated for 24 h with CS-PLGA nanoparticles (0.1 mg/mL) with surface-bound β-glucan at 0, 5, 10, 15, 20, or 25 ng. Jurkat cells were treated with 5% macrophage conditioned medium (respective nanoparticles doses) for 24 h (Supplementary Figure S4). At 24 h, CXCL10 gene expression was significantly increased at 5% 10 ng β-glucan conditioned medium (2.17 ± 0.33 TAI; *p* = 0.02), 5% 15 ng β-glucan conditioned medium (2.00 ± 0.22 TAI; *p* = 0.03), 5% 20 ng β-glucan conditioned medium (2.08 ± 0.42 TAI; *p* = 0.03), and 5% 25 ng β-glucan conditioned medium (3.01 ± 0.32 TAI; *p* ≤ 0.0001) compared to control (1.00 TAI) (Supplementary Figure S4A). At 24 h, IL-10 gene expression was significantly increased at 5% 10 ng β-glucan conditioned medium (1.94 ± 0.22 TAI; *p* = 0.018), 5%, 15 ng β-glucan conditioned medium (2.15 ± 0.24 TAI; *p* = 0.004), 5%, 20 ng β-glucan conditioned medium (2.18 ± 0.36 TAI; *p* = 0.006), and 5%, 25 ng β-glucan conditioned medium (2.37 ± 0.25 TAI; *p* = 0.008) compared to control (1.00 TAI) (Supplementary Figure S4B). At 24 h, IFNγ gene expression was significantly increased at 5%, 5 ng β-glucan conditioned medium (2.90 ± 0.05 TAI; *p* ≤ 0.0001), 5%, 10 ng β-glucan conditioned medium (1.88 ± 0.008 TAI; *p* ≤ 0.0001), 5%, 15 ng β-glucan conditioned medium (1.66 ± 0.03 TAI; *p* = 0.0005), 5%, 20 ng β-glucan conditioned medium (2.44 ± 0.45 TAI; *p* ≤ 0.0001), and 5%, 25 ng β-glucan conditioned medium (2.15 ± 0.24 TAI; *p* ≤ 0.0001) compared to control (1.00 TAI) (Supplementary Figure S4C).

#### Effect of surface bound β-glucan on survival of *Mtb* in macrophage

**FIGURE 5:**
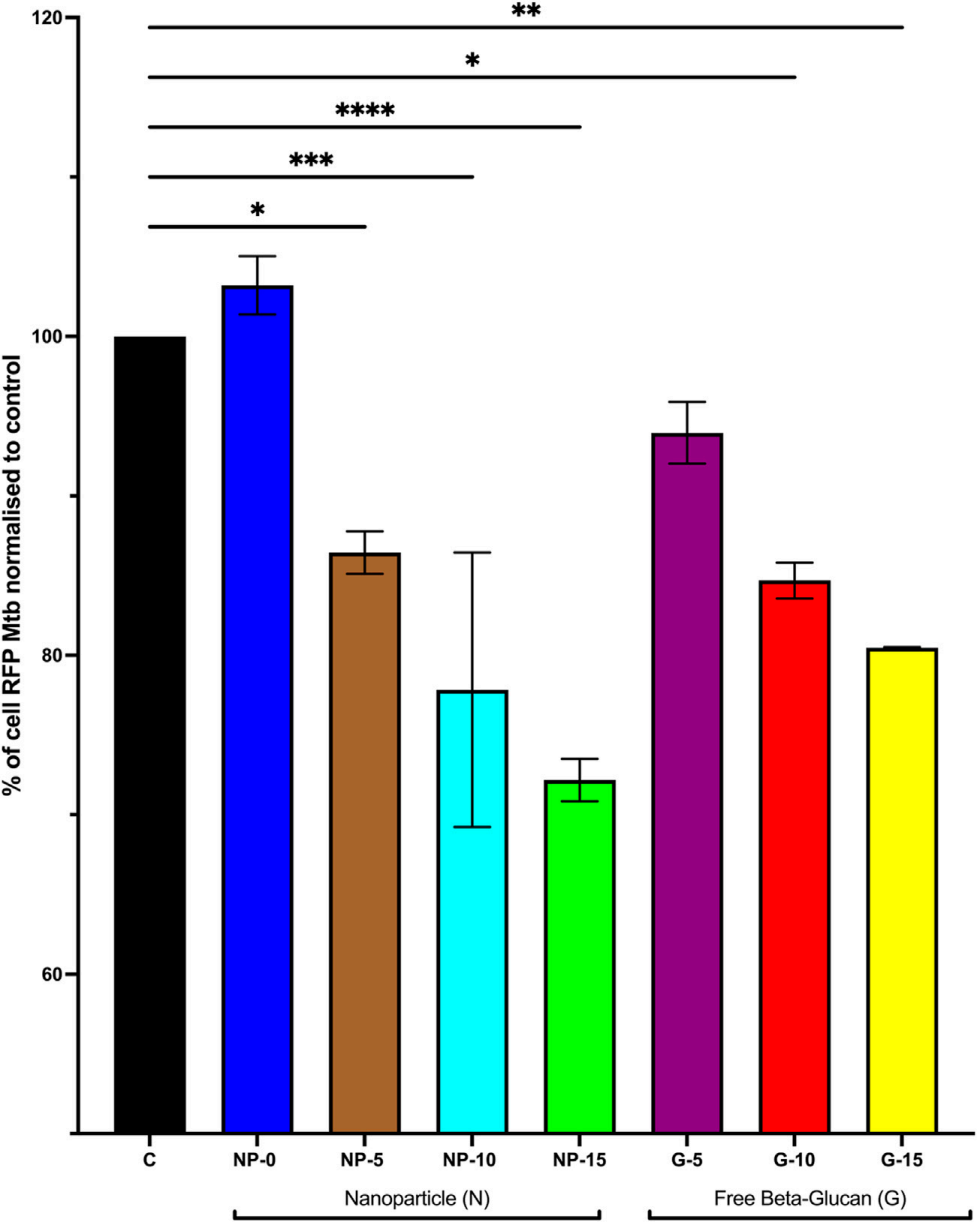
Effect of Increasing Amounts of Surface Bound β-glucan on Survival of Mtb in Macrophage. Monocyte derived macrophage (MDM) were infected with RFP-expressing *Mtb* (strain H37Ra) at TCID_50_ for 12 h. Cells were washed and then treated with CS-PLGA nanoparticles (0.1 mg/mL) with surface-bound β-glucan at 0, 5, 10, or 15 ng or free β-glucan at 5, 10, or 15 ng/ml for 5 days (*n* = 3–7). Cells were then harvested and fixed then analyzed by flow cytometry for % positive of RFP cells (indicative of presence of intracellular TB), normalized data, control set to 100%. Data shown represent the mean ± SEM. *p≤ 0.05; **p≤ 0.01; ***p≤ 0.001; ****p≤ 0.0001, comparison to control. C = control; N = nanoparticle; G = β-glucan.

Based on the findings described above (cytokine expression, cytokine secretion and ROS production), we determined the efficacy of the nanoparticle with surface bound β-glucan on *Mtb* survival in MDM or free β-glucan. MDM were infected with RFP-expressing *Mtb* (strain H37Ra) followed by treatment for 5 days with CS-PLGA nanoparticles (0.1 mg/mL) with surface-bound β-glucan at 0, 5, 10, or 15 ng or free β-glucan at 5, 10, or 15 ng/mL. Percentage of cell positive for RFP-*Mtb* was analyzed by flow cytometry (Figure 5). Data are represented as % of cells with RFP-*Mtb*. Data was normalized, and control was set to 100. Data demonstrate the intracellular accumulation of *Mtb* was significantly decreased at nanoparticles with 5 ng (86.44% ± 1.33%; *p* ≤ 0.05), 10 ng (77.83% ± 8.61%; *p* ≤ 0.001), or 15 ng (77.83± 1.33%; *p* ≤ 0.0001) surface bound β-glucan compared to the *Mtb* infection alone (100%). CS-PLGA nanoparticles (0.1 mg/mL) with no surface-bound β-glucan have no effect on the % of cells with RFP-*Mtb.* Free β-glucan significantly reduced the % of cells with RFP-*Mtb* at 10 (84.69% ± 1.13%, *p* ≤ 0.05) and 15 (80.47% ± 0.05%, *p* ≤ 0.01) ng/mL.

#### *In vivo* testing in healthy mice

**FIGURE 6:**
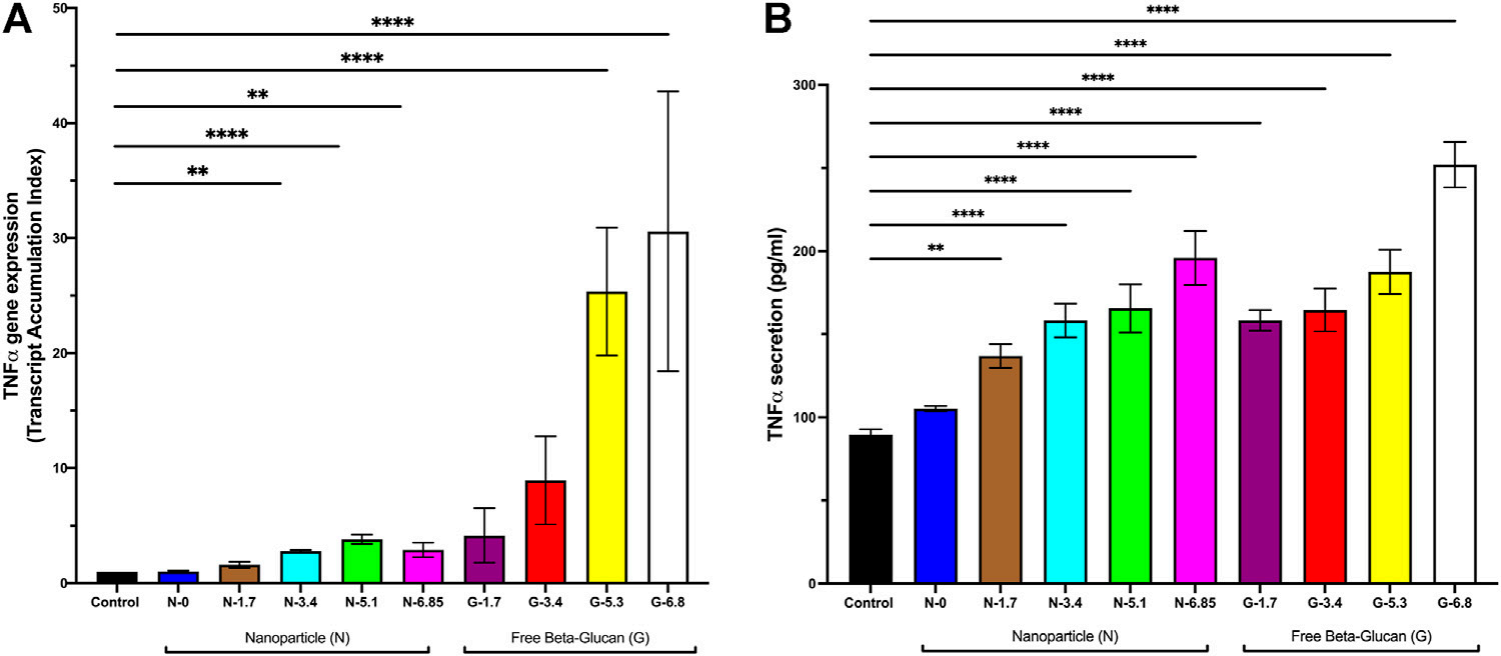
Effect of Increasing Amounts of Surface Bound β-glucan on mouse lungs. CD1 mice received CS-PLGA nanoparticles with surface bound β-glucan concentrations at 7.8, 15.6, or 31.3 ng β-glucan/gBW, or free Glu at 7.8, 15.6, or 31.3 ng β-glucan/gBW. BAL was performed 24 h post OPA. RNA was isolated from alveolar macrophage (isolated in BAL pellet) followed by analysis of TNFα gene expression. BAL supernatant was analyzed for TNFα secretion. **(A)** TNFα gene expression in alveolar macrophage following 24 h exposure (*n* = 3–8). **(B)** TNFα secretion 2 following 24 h exposure (*n* = 4–15). Data shown represent the mean ± SEM. **p ≤ 0.01; ***p ≤ 0.001; ****p ≤ 0.0001; comparison to control. N = nanoparticle; G = β-glucan.

We used our *in vitro* findings to inform *in vivo* dosing of Glu-CS-PLGA nanoparticles in healthy CD1 mice. Mice received nanoparticles with 0, 1.7, 3.4, 5.1 or 6.85 ng β-glucan/gBW or free β-glucan at 1.7, 3.4, 5.1 or 6.85 ng β-glucan/gBW by OPA, correlating with *in vitro* concentrations. BAL was performed 24 h post-OPA to collect alveolar macrophage and lungs were also harvested. TNFα gene expression was significantly increased with 3.4 ng β-glucan/gBW (2.81 ± 0.09 TAI; *p* ≤ 0.01), 5.1 ng β-glucan/gBW (3.83 ± 0.4 TAI; *p* ≤ 0.0001), and 6.85 ng β-glucan/gBW (2.89 ± 0.62 TAI; *p* ≤ 0.01) CS-PLGA nanoparticles 24 h post-OPA in alveolar macrophage (Figure 6A). CS-PLGA nanoparticles (0.1 mg/mL) with no surface-bound β-glucan have no effect on TNFα gene expression*.* Free β-glucan significantly increased TNFα gene expression at 5.1 ng β-glucan/gBW (25.37 ± 5.55 TAI, *p* ≤ 0.0001), and 6.85 ng β-glucan/gBW (30.59 ± 12.16 TAI, *p* ≤ 0.0001) ng/mL in alveolar macrophage. The gene expression of TNFα in total lung homogenates was unchanged (data not shown). TNFα protein secretion in the BAL was increased at 1.7 ng β-glucan/gBW (137.0 ± 7.13 pg/mL; *p* ≤ 0.001), 3.4 ng β-glucan/gBW (158.30 ± 10.09 pg/mL; *p* ≤ 0.0001), 5.1 ng β-glucan/gBW (165.6 ± 14.31 pg/mL; *p* ≤ 0.0001) and 6.85 ng β-glucan/gBW (198.6 ± 16.40 pg/mL; *p* ≤ 0.0001) nanoparticles 24 h post-OPA compared to control (89.72 ± 3.17 pg/mL) (Figure 6B). CS-PLGA nanoparticles (0.1 mg/mL) with no surface-bound β-glucan have no effect on TNFα protein secretion. Free β-glucan significantly increased TNFα protein secretion in the BAL at 1.7 ng β-glucan/gBW (158.30 ± 6.21 pg/mL; *p* ≤ 0.0001), 3.4 ng β-glucan/gBW (164.60 ± 12.89 pg/mL; *p* ≤ 0.0001), 5.1 ng β-glucan/gBW (187.50 ± 13.37 pg/mL; *p* ≤ 0.0001) and 6.85 ng β-glucan/gBW (252.01 ± 13.65 pg/mL; *p* ≤ 0.0001).

#### Effect of surface bound β-glucan on behavioral modifications in mice

We used a validated murine sepsis score with high specificity and sensitivity for predicting the onset of severe sepsis and death during the experimental timeline (Shrum et al., 2014). This model evaluates seven clinical variables: 1) appearance, 2) level of consciousness, 3) activity, 4) response to stimuli, 5) eyes, 6) respiration rate, and 7) respiration quality. The Murine Sepsis Score (MSS) rates on spontaneous activity, response to touch and auditory stimuli, posture, respiration rate and quality (labored breathing or gasping), and appearance. Greater than 21, indicates sepsis (Shrum et al., 2014). CD1 mice were dosed with OPA nanoparticles with surface bound glucan of 1.71, 3.43, 5.14, or 6.85 ng β-glucan/gBW. Free β-glucan was given at 1.71, 3.43, 5.14, or 6.85 ng β-glucan/gBW. Minimal changes occurred in the MSS in all experimental conditions; mice that received 6.85 ng free β-glucan experienced patches of hair piloerected and had slightly suppressed activity (Table 2).

**TABLE 2:** Effect of Surface Bound Glu on Behavioral Modifications in Mice. The Murine Sepsis Score (MSS) rates on spontaneous activity, response to touch and auditory stimuli, posture, respiration rate and quality (labored breathing or gasping), and appearance. Greater than 21, indicates sepsis. CD1 mice were given nanoparticles with 1.71, 3.43, 5.14 or 6.85 ng β-glucan/gBW by OPA. Free β-glucan was given at 1.71, 3.43, 5.14, or 6.85 ng β-glucan/gBW by OPA. Immediately before euthanasia animals were rated using the MSS. Data represent ±SEM. NP = Glu-CS-PLGA nanoparticles; G = free glucan.

	Murine sepsis score
C	0 ± 0
NP-0	0 ± 0
NP-5	0 ± 0
NP-10	0 ± 0
NP-15	0 ± 0
G-5	0 ± 0
G-10	0 ± 0
G-15	2 ± 0.28 ****

## Discussion

This study tested the hypothesis that nano-formulations designed to target macrophages can modulate the immune response and activate downstream pathways that counteract the suppressive effects of *Mtb*. In particular, we explored the impact of the ligand, β-glucan, on CS-PLGA nanoparticles on immunostimulatory properties both in a vitro and *in vivo* setting. The main objectives of the study were to: 1) determine the effects of nanogram concentrations of β-glucan (when bound to nanoparticles or free) on induction of biological mediators in macrophages, T cells, and in alveolar macrophage *in vivo*. Previous studies have only focused on free β-glucan at microgram concentrations; 2) determine the *in vitro* therapeutic efficacy of nanogram concentrations of β-glucan (when bound to nanoparticles or free) in reducing intracellular *Mtb* in macrophages. Previous studies have yielded conflicting findings on the therapeutic efficacy of microgram concentrations of β-glucan against *Mtb*; 3) determine the immunomodulatory potential of nanogram concentrations of β-glucan (when bound to nanoparticles or free) when given by OPA. Previous studies have focused only on oral, intraperitoneal, or intravenous administration of β-glucan; and 4) determine the overall *in vivo* safety and tolerability in mice following OPA of nanogram concentrations of β-glucan (when bound to nanoparticles or free). This is the first study to examine this Glu-CS-PLGA nanoparticle formulation *in vivo* and free β-glucan at nanogram concentrations.

We were particularly interested in the targeting the macrophage, which is an innate antigen-presenting immune cell and the host cell to *Mtb* (Weiss and Schaible, 2015; Upadhyay et al., 2018). When designing our nanoparticle, we chose to use β-glucan as our ligand, as it is a polysaccharide that is a fundamental structural component of yeast and mold cell walls. According to Riggi and Di Luzio’s 1961 research, β-glucan is an immunomodulator that activates complement, stimulates the innate immune system, and possesses anti-tumor and anti-infection properties. Insoluble β-glucan (particulate) has been shown to primarily interact with the dectin-1 receptor on dendritic cells (DC) and macrophage. It induces phagocytosis and T-cell differentiation. While soluble β-glucan binds to CR3 which activates the complement system (Synytsya and Novak, 2014; Mirza et al., 2017; Pedro et al., 2021). Hetland and Sandven’s (2002) study discovered that microgram concentrations of β-glucan reduced *Mtb* H37Rv survival in BALB/c mice’s peritoneal macrophages, with particulate β-glucan having more of an effect than soluble β-glucan (Hetland and Sandven, 2002). Furthermore, Moorlag et al., in 2020 found that training with microgram quantities of β-glucan significantly lowered the number of Mtb colony-forming units compared to the control, indicating that β-glucan has anti-mycobacterial effects (Moorlag et al., 2020). They also demonstrate that mice given an intraperitoneal injection of β-glucan had significantly lower Mtb burden in the lung compared to PBS-control (Moorlag et al., 2020). In contrast, Betz et al. found that at microgram concentrations of insoluble whole glucan particles and a soluble glucan extract did not impede intracellular *Mtb* growth in macrophages (Betz et al., 2011). Furthermore, most *in vivo* studies have given both soluble and particulate β-glucan by oral, intravenous or intraperitoneal injection and the majority of research has focused on the priming effects of β-glucan on the immune system (Vetvicka et al., 2020) or prophylaxis treatment (Betz et al., 2011). In human subjects, oral administration of β-glucan to healthy subjects have shown no adverse events, however studies found that there was no systemic adsorption of β-glucan. Furthermore, Leentjens et al. found that oral administration of β-glucan for 7 days did not induce serum cytokine production (Leentjens et al., 2014). Authors conclude that the use of oral β-glucan to enhance innate immune responses was not supported. This suggests that β-glucan should be targeted to specific organs or tissues for a localized response. Furthermore, inhaled therapies for *Mtb,* which target therapies to the lungs are needed. Therefore, we designed our CS-PLGA nanoparticle with β-glucan as our targeting ligand based on the studies above. In terms of translation, we envision this therapy as an adjuvant inhalational TB therapy, which would be a more feasible and practical approach for human drug delivery.

We investigated the effect of surface-bound β-glucan on the size of CS-PLGA nanoparticles. β-glucan is a heterogeneous carbohydrate composed of D-glucose monomers that polymerize through linear 1-3-glycosidic linkages (Synytsya and Novak, 2014). We wanted to determine if changing the surface concentration would influence the nanoparticle size. Our data demonstrate that the surface-bound concentrations studied did not result in any changes in the mean nanoparticle size. Epi-fluorescence imaging following exposure to CS-PLGA nanoparticles with surface-bound β-glucan at 5, 10, 15, 20, or 25 ng demonstrated that the maximum amount of intracellular uptake occurred at 15 ng of surface-bound β-glucan. Additionally, cellular viability dropped significantly as the concentration of surface-bound β-glucan increased. CS-PLGA nanoparticles with 20 and 25 ng of surface-bound β-glucan dramatically reduced THP-1 macrophage viability after 24-and 48-h post-incubation. Cellular viability also decreased at the higher concentrations of free β-glucan supporting the role of β-glucan bound to CS-PLGA nanoparticles as a safer adjuvant therapeutic.

Previous studies have shown that free β-glucan at microgram concentrations elicits an *in vitro* cytokine response in macrophage (Hetland and Sandven, 2002; Betz et al., 2011). Our study investigated the effects on nanogram concentrations of surface bound β-glucan. We found that cytokine gene expression, TNFα protein secretion, and ROS generation increased at 5–25 ng amounts of β-glucan whether bound to the nanoparticle or in free form. While at our study was at a significantly lower concentration, we confirmed previous studies that demonstrate the innate immune activation of free β-glucan and Glu-CS-PLGA nanoparticles (Hetland and Sandven, 2002; Betz et al., 2011). Since β-glucan is a PAMP that binds to the Dectin-1 PRR on macrophages (Uribe-Querol and Rosales, 2020), we aimed to establish that the effects of surface-bound β-glucan on cytokine gene expression were mediated through the Dectin-1 receptor. Our results demonstrate that the increase in IL-1β, IL-6, and TNFα gene expression induced by β-glucan was inhibited in the presence of laminarin, a Dectin-1 antagonist. This suggests that the Glu-CS-PLGA nanoparticle activates downstream pathways by binding Dectin-1 receptors. Furthermore, our data demonstrate a biphasic response in cytokine gene expression, cytokine secretion, and ROS production. This may be primarily due to the reduced cell viability observed at 20 and 25 ng of surface-bound β-glucan. However, it may also be due to the downregulation of the Dectin-1 receptor, which restricts further immune response by reducing Dectin-1 receptor expression at higher concentrations of surface-bound β-glucan (20 ng or 25 ng). A study showed that a high-affinity synthetic PPAR-γ ligand, created during the late phase of inflammation, blocks Dectin-1 activation by interfering with the CARD9, mitogen-activated protein kinase, and nuclear factor-kB signaling pathways, confirming their significance as negative-feedback regulators of potentially harmful inflammatory responses (Kock et al., 2011). These findings correspond with our results, suggesting that increasing the concentration of β-glucan likely downregulates the receptor. Future studies are necessary to further explore this concept.

Macrophage and T cells play an integral role in the immune response, both exerting a paracrine response on each other. When a macrophage is activated, it secretes pro-inflammatory mediators such as TNFα, IL-1, IL-6, IL-8, and IL-12. The cytokines secreted by macrophage, in particular IL-12, activate surrounding T helper (CD4^+^) cells (Arango Duque and Descoteaux, 2014). Activated T cells then produced IFNγ, TNFα, IL-10, and CXCL10. IFNγ then acts on macrophage to induce nitric oxide synthase, associated with the production of ROS in macrophage and it supports phagosome maturation (Mayer-Barber and Barber, 2015; Zhai et al., 2019; Maphasa et al., 2020; Uribe-Querol and Rosales, 2020). These mechanisms are suppressed by *Mtb*. Since T cells play an important role, we sought to determine the Glu-CS-PLGA effects on T cells. Not surprisingly we found no significant changes in cytokine gene expression following direct incubation with nanoparticles with increasing concentrations of surface bound β-glucan. The lack of response is due primarily to the lack of expression of Dectin-1 receptors on T cells and that T cells are not phagocytes (Serezani et al., 2012). Since we saw no direct effect on T cells, we proposed a culture medium transfer experiment. In our transfer study, macrophage conditioned media was given to T cells. Conditioned macrophage medium was defined as cell supernatant collected from THP-1 macrophage that were incubated for 24 h with CS-PLGA nanoparticles with increasing concentrations of surface bound β-glucan. Exposure of T cells to macrophage conditioned medium activated T cells as demonstrated by the increase gene expression of CXCL10, IL-10, and IFN chemokine/cytokine production. As described above, the biological mediators produced by macrophage, in particular IL-12, is likely the cause of T cell activation (Uribe-Querol and Rosales, 2020). Future studies are necessary that prevent the secretion of IL-12 or block IL-12 from macrophage to determine if this is the biological mediator that is elicited by our nanoparticle.

Previous studies have yielded conflicting results regarding the effects of β-glucan on intracellular *Mtb* growth in macrophage *in vitro*. Hetland and Sandven’s (2002) study discovered that microgram concentrations of β-glucan reduced Mtb H37Rv survival in BALB/c mice’s peritoneal macrophages, with particulate β-glucan having more of an effect than soluble β-glucan (Hetland and Sandven, 2002). In contrast, Betz et al. found that at microgram concentrations, insoluble whole glucan particles (WGP) and a soluble glucan extract did not impede intracellular *Mtb* CFU in macrophages (Betz et al., 2011). Conflicting results on efficacy may have been due to the experimental approach and the sources of β-glucan. Our studies found that 10 and 15 nanogram concentrations of free β-glucan and our Glu-CS-PLGA nanoparticles (surface bound β-glucan at 5, 10, and 15 ng) significantly reduced macrophage percent positive for RFP-*Mtb*. This study is the first study to show that nanogram concentrations of free β-glucan and our nanoparticle have efficacy against *Mtb*. These findings support our previous findings that Glu-CS-PLGA nanoparticles inhibit *mycobacterium* (Kutscher et al., 2019) as well as other studies demonstrating the anti-mycobacterial effects of β-glucan (Hetland and Sandven, 2002; Khan et al., 2020; Moorlag et al., 2020). Furthermore, this study is the first study to demonstrate Glu-CS-PLGA nanoparticles alone, without drug encapsulation, reduce *Mtb* growth demonstrating the potential adjuvant role of these nanoparticles. Additionally, when comparing Glu-CS-PLGA nanoparticles to free β-glucan, the nanoparticles had increased efficacy compared to free β-glucan. This supports the role of our nanoparticle as a strong inhaled adjuvant compared to inhaled free β-glucan. While our data conflicts with Betz et al. that demonstrates no effect of β-glucan on intracellular *Mtb* in macrophages (Betz et al., 2011); our findings are likely different due to the strain of *Mtb* studied.

Our goal in developing immunostimulatory nanoparticles is to enhance the innate immune response by promoting cytokine production and ROS species, but it is important to note that excessive immune activation can be harmful. TB-Immune Reconstitution Inflammatory Syndrome (IRIS) is an example of an excessive immune response against Mtb that can occur in HIV-infected or uninfected patients during or after anti-TB therapy, resulting in poor outcomes (Lanzafame and Vento, 2016). Since most TB patients with HIV are on ART, it is important to consider the potential for TB-IRIS when developing new therapies for TB and HIV-1. We used our *in vitro* findings to inform dosing for *in vivo* studies in healthy mice, taking TB-IRIS into account. Our data showed that localized inflammatory responses occurred in alveolar macrophages with increases in TNFα gene expression and protein expression. This localized response has the potential to promote clearance of intracellular pathogens by increasing cytokines and ROS in the macrophage milieu. Moreover, TNFα gene expression in total lung homogenates and TNFα protein secretion in serum were not increased, indicating that Glu-CS-PLGA nanoparticles did not induce a systemic inflammatory response or cytokine storm within the entire lung. All nanoparticles and free β-glucan stimulated the release of TNFα in the range of 500–2000 pg/mL, which is similar to TNFα concentrations in a TB mouse model after drug treatment (Maiga et al., 2015). Accumulation of albumin in the interstitial and alveolar spaces indicates damage to blood vessels or the alveolar epithelium (Beretta et al., 2021), but we found that administration of Glu-CS-PLGA nanoparticles at all doses did not increase albumin concentrations in the BAL, suggesting that damage to the alveolar epithelium did not occur. These findings demonstrate that inhalational delivery of CS-PLGA nanoparticles is safe and tolerable. Furthermore, using a validated murine sepsis score that accurately predicts the onset of sepsis we found that 24 h post exposure to Glu-CS-PLGA nanoparticles had no effect on behavioral aspects of mice. Mice that received free β-glucan at 6.85 ng/gBW experienced patches of hair piloerected and had slightly suppressed activity further supporting the role of the Glu-CS-PLGA over free β-glucan as an adjuvant therapy.

In conclusion, our study indicates that Glu-CS-PLGA nanoparticles are a promising immunostimulatory tool for the treatment of TB. Our data demonstrate that Glu-CS-PLGA nanoparticles stimulate macrophages through Dectin-1 receptors and activate T cells via a paracrine effect *in vitro* at nanogram concentrations. Additionally, we show that these nanoparticles effectively reduce the percentage of RFP-*Mtb* positive macrophages. We also demonstrate that when given by OPA, Glu-CS-PLGA nanoparticles elicit a cytokine response that is limited to alveolar macrophage. Furthermore, no adverse effects in the alveolar epithelium occurred. Data support preliminary safety and feasibility of Glu-CS-PLGA nanoparticle as an adjuvant therapy for *Mtb.* Collectively, our findings provide a foundation for the development of a new adjuvant immunotherapy for TB, with further studies needed to establish the full safety profile and optimize dosing for clinical translation.

## Data Availability

The raw data supporting the conclusion of this article will be made available by the authors, without undue reservation.

## References

[B1] Alluri R. H. L. Kutscher B. A. Mullan B. A. Davidson B. A. Knight P. R. (2017). Open tracheostomy gastric acid aspiration murine model of acute lung injury results in maximal acute nonlethal lung injury. J. Vis. Exp. 120, 54700. 10.3791/54700 PMC5405730 28287530

[B2] Arango Duque G. Descoteaux A. (2014). Macrophage cytokines: Involvement in immunity and infectious diseases. Front. Immunol. 5, 491. 10.3389/fimmu.2014.00491 25339958 PMC4188125

[B3] Armstrong J. A. Hart P. D. (1971). Response of cultured macrophages to *Mycobacterium tuberculosis*, with observations on fusion of lysosomes with phagosomes. J. Exp. Med. 134 (3), 713–740. 10.1084/jem.134.3.713 15776571 PMC2139093

[B4] Barker R. Patrick H. (1997). Tuberculosis--impact of the HIV epidemic. S Afr. Med. J. 87 (10), 1384. 9472255

[B5] Beretta E. Romano F. Sancini G. Grotberg J. B. Nieman G. F. Miserocchi G. (2021). Pulmonary interstitial matrix and lung fluid balance from normal to the acutely injured lung. Front. Physiol. 12, 781874. 10.3389/fphys.2021.781874 34987415 PMC8720972

[B6] Betz B. E. Azad A. K. Morris J. D. Rajaram M. V. S. Schlesinger L. S. (2011). β-Glucans inhibit intracellular growth of Mycobacterium bovis BCG but not virulent *Mycobacterium tuberculosis* in human macrophages. Microb. Pathog. 51 (4), 233–242. 10.1016/j.micpath.2011.06.006 21762773 PMC4266430

[B7] Bustin S. A. (2002). Quantification of mRNA using real-time reverse transcription PCR (RT-PCR): Trends and problems. J. Mol. Endocrinol. 29 (1), 23–39. 10.1677/jme.0.0290023 12200227

[B8] Carr W. Kurbatova E. Starks A. Goswami N. Allen L. Winston C. (2022). Interim guidance: 4-Month rifapentine-moxifloxacin regimen for the treatment of drug-susceptible pulmonary tuberculosis - United States, 2022. MMWR Morb. Mortal. Wkly. Rep. 71 (8), 285–289. 10.15585/mmwr.mm7108a1 35202353

[B9] Chanput W. Mes J. J. Wichers H. J. (2014). THP-1 cell line: An *in vitro* cell model for immune modulation approach. Int. Immunopharmacol. 23 (1), 37–45. 10.1016/j.intimp.2014.08.002 25130606

[B10] Chomczynski P. Sacchi N. (2006). The single-step method of RNA isolation by acid guanidinium thiocyanate-phenol-chloroform extraction: Twenty-something years on. Nat. Protoc. 1 (2), 581–585. 10.1038/nprot.2006.83 17406285

[B11] Dube A. Reynolds J. L. Law W. C. Maponga C. C. Prasad P. N. Morse G. D. (2014). Multimodal nanoparticles that provide immunomodulation and intracellular drug delivery for infectious diseases. Nanomedicine 10 (4), 831–838. 10.1016/j.nano.2013.11.012 24333593

[B12] Essaji Y. Yang Y. Albert C. J. Ford D. A. Brown R. J. (2013). Hydrolysis products generated by lipoprotein lipase and endothelial lipase differentially impact THP-1 macrophage cell signalling pathways. Lipids 48 (8), 769–778. 10.1007/s11745-013-3810-6 23794138 PMC4727182

[B13] Frehel C. de Chastellier C. Lang T. Rastogi N. (1986). Evidence for inhibition of fusion of lysosomal and prelysosomal compartments with phagosomes in macrophages infected with pathogenic *Mycobacterium avium* . Infect. Immun. 52 (1), 252–262. 10.1128/iai.52.1.252-262.1986 2870027 PMC262228

[B14] Hetland G. Sandven P. (2002). β-1,3-Glucan reduces growth of *Mycobacterium tuberculosis* in macrophage cultures. FEMS Immunol. Med. Microbiol. 33 (1), 41–45. 10.1016/s0928-8244(01)00312-1 11985967

[B15] Khan S. H. Khan S. Hashim N. Khan I. (2020). β-1,3-glucan attenuated chronic unpredictable mild stress-induced cognitive impairment in rodents via normalizing corticosterone levels. Cent. Nerv. Syst. Agents Med. Chem. 20 (3), 206–217. 10.2174/1871524920666200810142359 32778039

[B16] Kock G. Bringmann A. Held S. A. Daecke S. Heine A. Brossart P. (2011). Regulation of dectin-1-mediated dendritic cell activation by peroxisome proliferator-activated receptor-gamma ligand troglitazone. Blood 117 (13), 3569–3574. 10.1182/blood-2010-08-302224 21296999

[B17] Kutscher H. L. Morse G. D. Prasad P. N. Reynolds J. L. (2019). *In vitro* pharmacokinetic cell culture system that simulates physiologic drug and nanoparticle exposure to macrophages. Pharm. Res. 36 (3), 44. 10.1007/s11095-019-2576-9 30710170 PMC6547366

[B18] Lanzafame M. Vento S. (2016). Tuberculosis-immune reconstitution inflammatory syndrome. J. Clin. Tuberc. Other Mycobact. Dis. 3, 6–9. 10.1016/j.jctube.2016.03.002 31723680 PMC6850228

[B19] Leentjens J. Quintin J. Gerretsen J. Kox M. Pickkers P. Netea M. G. (2014). The effects of orally administered Beta-glucan on innate immune responses in humans, a randomized open-label intervention pilot-study. PLoS One 9 (9), e108794. 10.1371/journal.pone.0108794 25268806 PMC4182605

[B20] Liu C. H. Liu H. Ge B. (2017). Innate immunity in tuberculosis: Host defense vs pathogen evasion. Cell. Mol. Immunol. 14 (12), 963–975. 10.1038/cmi.2017.88 28890547 PMC5719146

[B21] MacMicking J. D. Taylor G. A. McKinney J. D. (2003). Immune control of tuberculosis by IFN-gamma-inducible LRG-47. Science 302 (5645), 654–659. 10.1126/science.1088063 14576437

[B22] Maiga M. Ahidjo B. A. Maiga M. C. Cheung L. Pelly S. Lun S. (2015). Efficacy of adjunctive tofacitinib therapy in mouse models of tuberculosis. EBioMedicine 2 (8), 868–873. 10.1016/j.ebiom.2015.07.014 26425693 PMC4563140

[B23] Maphasa R. E. Meyer M. Dube A. (2020). The macrophage response to *Mycobacterium tuberculosis* and opportunities for autophagy inducing nanomedicines for tuberculosis therapy. Front. Cell. Infect. Microbiol. 10, 618414. 10.3389/fcimb.2020.618414 33628745 PMC7897680

[B24] Mayer-Barber K. D. Barber D. L. (2015). Innate and adaptive cellular immune responses to *Mycobacterium tuberculosis* infection. Cold Spring Harb. Perspect. Med. 5 (12), a018424. 10.1101/cshperspect.a018424 26187873 PMC4665043

[B25] Mirza Z. Soto E. R. Dikengil F. Levitz S. M. Ostroff G. R. (2017). Beta-glucan particles as vaccine adjuvant carriers. Methods Mol. Biol. 1625, 143–157. 10.1007/978-1-4939-7104-6_11 28584989

[B26] Moorlag S. Khan N. Novakovic B. Kaufmann E. Jansen T. van Crevel R. (2020). β-Glucan induces protective trained immunity against *Mycobacterium tuberculosis* infection: A key role for IL-1. Cell. Rep. 31 (7), 107634. 10.1016/j.celrep.2020.107634 32433977 PMC7242907

[B27] Nainwal N. Sharma Y. Jakhmola V. (2022). Dry powder inhalers of antitubercular drugs. Tuberc. (Edinb) 135, 102228. 10.1016/j.tube.2022.102228 35779497

[B28] Neun B. W. Cedrone E. Potter T. M. Crist R. M. Dobrovolskaia M. A. (2020). Detection of beta-glucan contamination in nanotechnology-based formulations. Molecules 25 (15), 3367. 10.3390/molecules25153367 32722261 PMC7436117

[B29] Pedro A. R. V. Lima T. Frois-Martins R. Leal B. Ramos I. C. Martins E. G. (2021). Dectin-1-Mediated production of pro-inflammatory cytokines induced by yeast beta-glucans in bovine monocytes. Front. Immunol. 12, 689879. 10.3389/fimmu.2021.689879 34122455 PMC8195389

[B30] Radonic A. Thulke S. Mackay I. M. Landt O. Siegert W. Nitsche A. (2004). Guideline to reference gene selection for quantitative real-time PCR. Biochem. Biophys. Res. Commun. 313 (4), 856–862. 10.1016/j.bbrc.2003.11.177 14706621

[B31] Ramachandra L. Smialek J. L. Shank S. S. Convery M. Boom W. H. Harding C. V. (2005). Phagosomal processing of *Mycobacterium tuberculosis* antigen 85B is modulated independently of mycobacterial viability and phagosome maturation. Infect. Immun. 73 (2), 1097–1105. 10.1128/iai.73.2.1097-1105.2005 15664953 PMC547092

[B32] Reynolds J. L. Law W. C. Mahajan S. D. Aalinkeel R. Nair B. Sykes D. E. (2012a). Morphine and galectin-1 modulate HIV-1 infection of human monocyte-derived macrophages. J. Immunol. 188 (8), 3757–3765. 10.4049/jimmunol.1102276 22430735 PMC3324598

[B33] Reynolds J. L. Law W. C. Mahajan S. D. Aalinkeel R. Nair B. Sykes D. E. (2012b). Nanoparticle based galectin-1 gene silencing, implications in methamphetamine regulation of HIV-1 infection in monocyte derived macrophages. J. Neuroimmune Pharmacol. 7 (3), 673–685. 10.1007/s11481-012-9379-7 22689223 PMC3419803

[B48] Riggi S. J. Di Luzio N. R. (1961). Identification of a reticuloendothelial stimulating agent in zymosan. Am. J. Physiol. 200, 297–300. 10.1152/ajplegacy.1961.200.2.297 13741617

[B34] Rio D. C. Ares M. Jr. Hannon G. J. Nilsen T. W. (2010). Purification of RNA using TRIzol (TRI reagent). Cold Spring Harb. Protoc. 2010 (6), pdb.prot5439. 10.1101/pdb.prot5439 20516177

[B35] Serezani C. H. Kane S. Collins L. Morato-Marques M. Osterholzer J. J. Peters-Golden M. (2012). Macrophage dectin-1 expression is controlled by leukotriene B4 via a GM-CSF/PU.1 axis. J. Immunol. 189 (2), 906–915. 10.4049/jimmunol.1200257 22696442 PMC3392366

[B36] Shi L. Jiang Q. Bushkin Y. Subbian S. Tyagi S. (2019). Biphasic dynamics of macrophage immunometabolism during *Mycobacterium tuberculosis* infection. mBio 10 (2), e02550. 10.1128/mbio.02550-18 30914513 PMC6437057

[B37] Shrum B. Anantha R. V. Xu S. X. Donnelly M. Haeryfar S. M. McCormick J. K. (2014). A robust scoring system to evaluate sepsis severity in an animal model. BMC Res. Notes 7, 233. 10.1186/1756-0500-7-233 24725742 PMC4022086

[B38] Smith A. J. Graves B. Child R. Rice P. J. Ma Z. Lowman D. W. (2018). Immunoregulatory activity of the natural product laminarin varies widely as a result of its physical properties. J. Immunol. 200 (2), 788–799. 10.4049/jimmunol.1701258 29246954 PMC5760317

[B39] Synytsya A. Novak M. (2014). Structural analysis of glucans. Ann. Transl. Med. 2 (2), 17. 10.3978/j.issn.2305-5839.2014.02.07 25332993 PMC4202478

[B40] Upadhyay S. Mittal E. Philips J. A. (2018). Tuberculosis and the art of macrophage manipulation. Pathog. Dis. 76 (4), fty037. 10.1093/femspd/fty037 29762680 PMC6251593

[B41] Uribe-Querol E. Rosales C. (2020). Phagocytosis: Our current understanding of a universal biological process. Front. Immunol. 11, 1066. 10.3389/fimmu.2020.01066 32582172 PMC7280488

[B42] Van Hoecke L. Job E. R. Saelens X. Roose K. (2017). Bronchoalveolar lavage of murine lungs to analyze inflammatory cell infiltration. J. Vis. Exp. 123, 55398. 10.3791/55398 PMC5607888 28518083

[B43] Vetvicka V. Vannucci L. Sima P. (2020). β-glucan as a new tool in vaccine development. Scand. J. Immunol. 91 (2), e12833. 10.1111/sji.12833 31544248

[B44] Weiss G. Schaible U. E. (2015). Macrophage defense mechanisms against intracellular bacteria. Immunol. Rev. 264 (1), 182–203. 10.1111/imr.12266 25703560 PMC4368383

[B45] WHO (2021a). Global tuberculosis report 2021. Geneva: WHO.

[B46] WHO (2021b). Tuberculosis.

[B47] Zhai W. Wu F. Zhang Y. Fu Y. Liu Z. (2019). The immune escape mechanisms of Mycobacterium tuberculosis. Int. J. Mol. Sci. 20 (2), 340. 10.3390/ijms20020340 30650615 PMC6359177

