# Supplementary material for: An immunoregulator nanomedicine approach for the treatment of tuberculosis
Source: Front Bioeng Biotechnol. 2023 May 25;11:1095926. doi: 10.3389/fbioe.2023.1095926 (PMC10249870; doi:10.3389/fbioe.2023.1095926)
Supplement: Supplementary file 1 [file DataSheet1.docx]

Supplementary Material

Article Title

Luona Yang ^1^, Lee Chaves ^2^, Hilliard L. Kutscher^1^, Shanta Karki ^1^, Maria Tamblin ^1^ , Patrick Kenney^3^, Jessica L. Reynolds^1*^

*** Correspondence:** Corresponding Author: jlr8@buffalo.edu

Supplementary Data

**
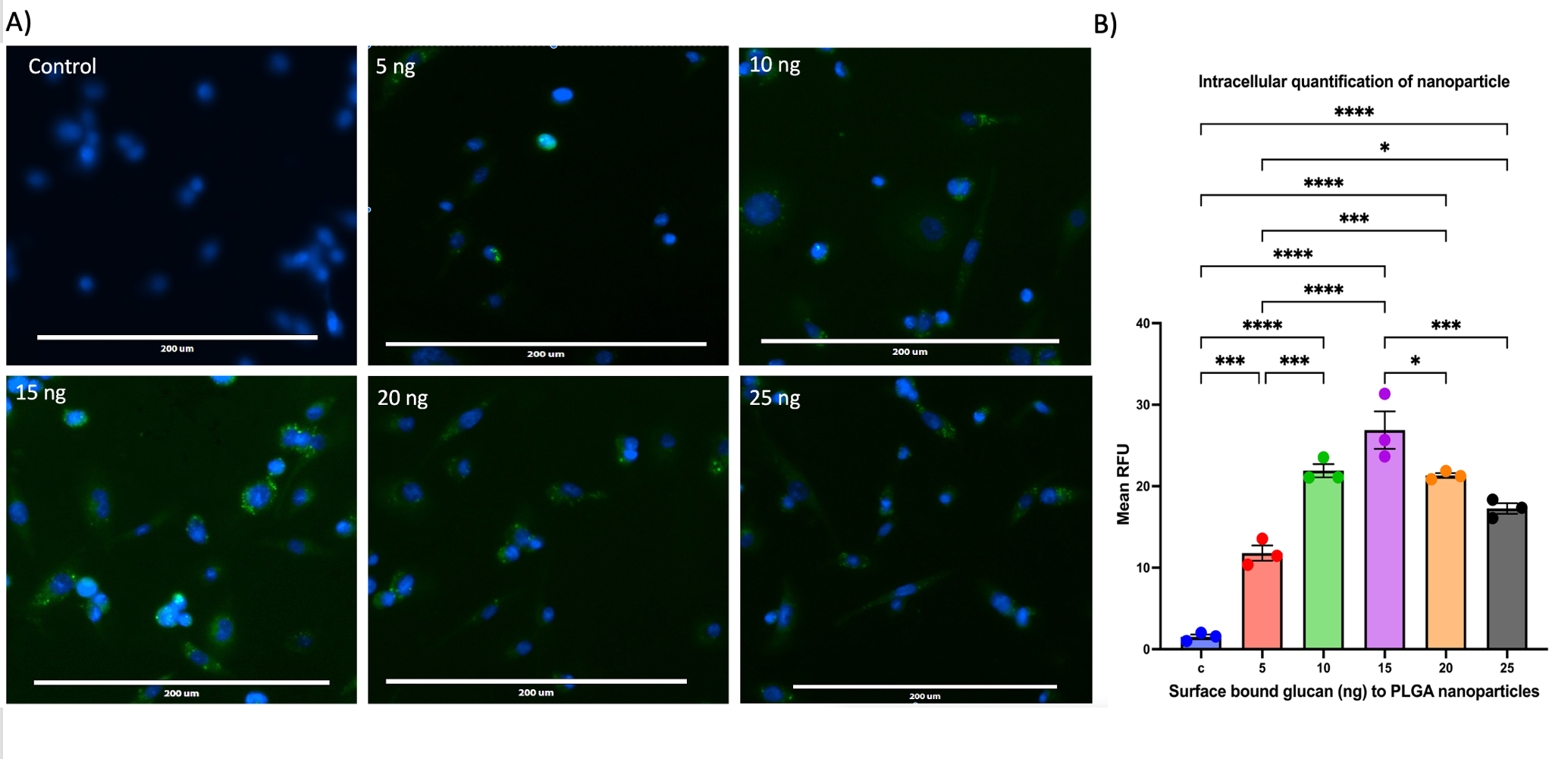
**

Figure S1. Intracellular Uptake in Macrophage of CS-PLGA Nanoparticles with Increasing Amounts of Surface Bound **β-glucan**. **A)** CS-PLGA nanoparticles (0.1 mg/ml) with surface-bound β-glucan at 5, 10, 15, 20, or 25 ng (Green color) were incubated with THP-1 macrophage for 24 hr (n=3). Cells were fixed and counterstained with DAPI (nuclear stain, blue color); then observed using epifluorescence (EVOS® FL Cell Imaging System. **B)** Quantification of intracellular relative fluorescence density (RFU) was done using ImageJ software. Data shown represent the mean ± SEM. *p≤0.05; ***p≤0.001; ****p≤ 0.0001; comparisons to control (c) or comparisons to the mean of each column with the mean of every other column.

Figure S2. Cellular Viability of Macrophage after Exposure to Nanoparticles and Free **β-glucan**. CS-PLGA nanoparticles (0.1 mg/ml) with surface-bound β-glucan at 0, 5, 10, 15, 20, or 25 ng or free β-glucan at 5, 10, 15, 20, or 25 ng/ml were incubated with THP-1 macrophage for 24 hr and 48 hr. Cellular viability was determined using a CyQUANT™ MTT Cell Viability Assay. **A)** 24 hr exposure (n=3 to 9). **B)** 48 hr exposure (n=3 to 9). Data shown represent the mean ± SEM. *p≤ 0.05; **p≤ 0.01; ***≤ 0.001; comparison to control (c). N = nanoparticle; G = β-glucan.

**
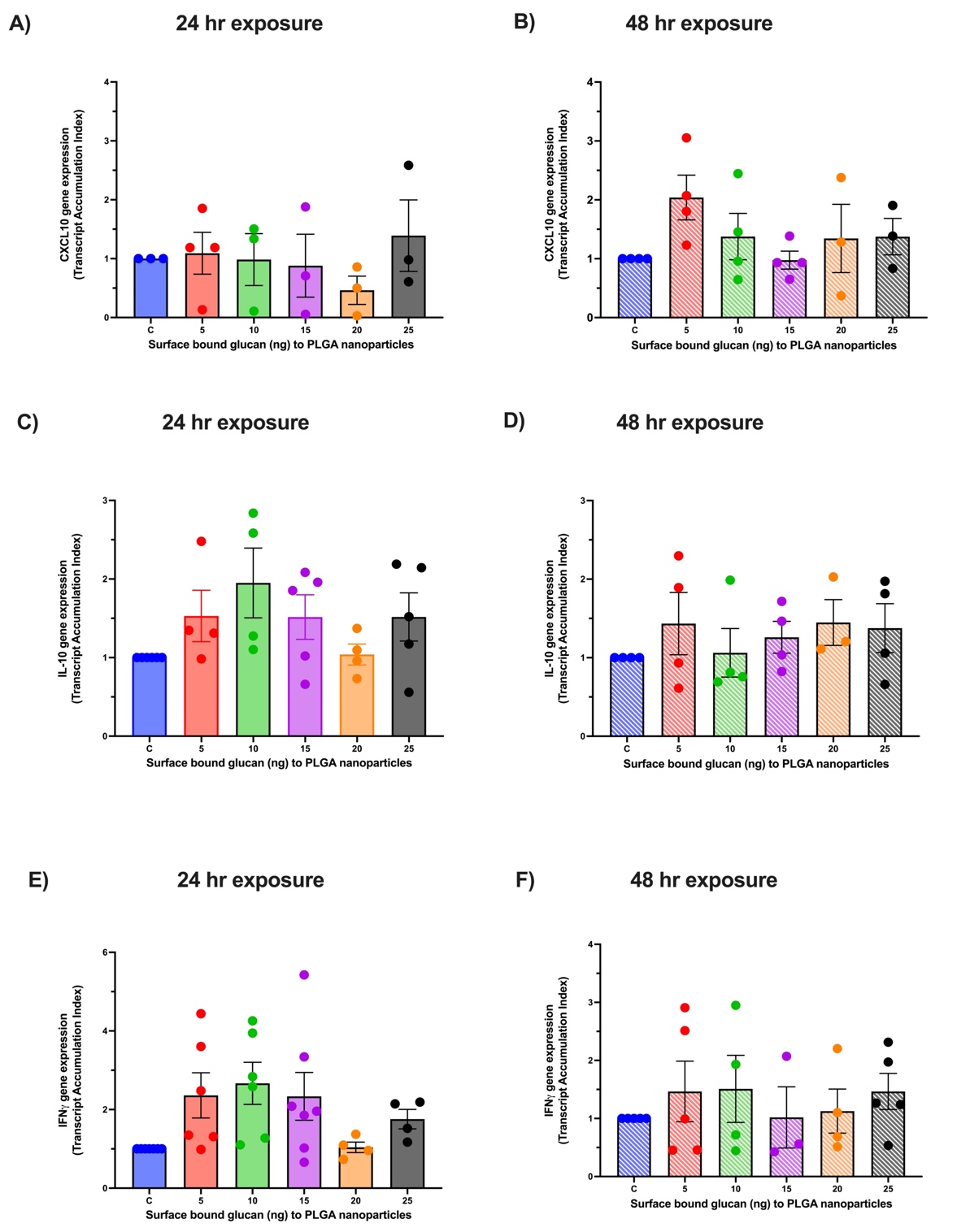
**

Figure S3. Effect of Increasing Amounts of Surface Bound **β-glucan** on Cytokine Gene Expression from T cells. CS-PLGA nanoparticles (0.1 mg/ml) with surface-bound β-glucan at 5, 10, 15, 20, or 25 ng were incubated with Jurkat T cells for 24 hr and 48 hr; RNA was isolated and changes in gene expression were analyzed using Q-PCR. **A)** CXCL10 gene expression following 24 hr exposure to nanoparticles (n = 5). **B)** CXCL10 gene expression following 48 hr incubation with nanoparticles (n=5). **C)** IL-10 gene expression following 24 hr exposure to nanoparticles (n=7). **D)** IL-10 gene expression following 48 hr incubation with nanoparticles (n = 5). **E)** IFNγ gene expression following 24 hr exposure to nanoparticles (n=7). **F)** IFNγ gene expression following 48 hr incubation with nanoparticles (n=5). Data shown represent the mean ± SEM. p=ns; comparison to control (c).

**
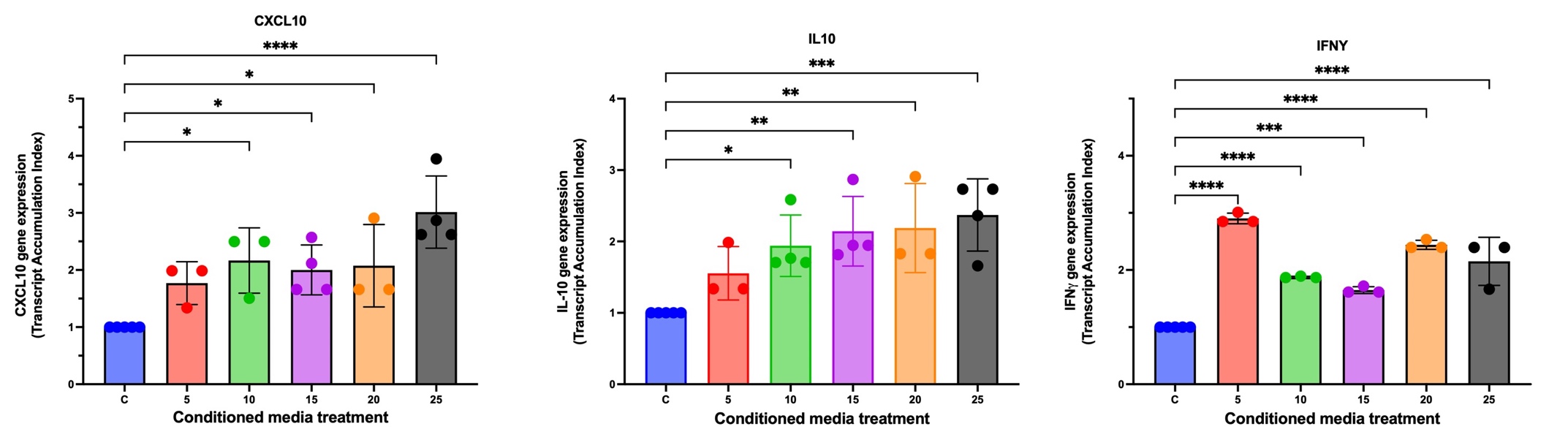
**

Figure S4. Effect of Increasing Amounts of Macrophage Conditioned Medium on Gene Expression from Jurkat T cells. Jurkat T cells were incubated with 5% of macrophage conditioned medium (supernatant from nanoparticle experiments in Figure 6) for 24 hr. **A)** CXCL10 gene expression (n = 5). **B)** IL-10 gene expression (n=5). **C)** IFNγ gene expression (n=5). Data shown represent the mean ± SEM. *p≤0.05; **p≤0.01; ***p≤0.001; ****p≤0.0001; comparison to control (c).
